# Evolutionary Processes Driving the Rise and Fall of *Staphylococcus aureus* ST239, a Dominant Hybrid Pathogen

**DOI:** 10.1128/mBio.02168-21

**Published:** 2021-12-14

**Authors:** Jacqueline L. Gill, Jessica Hedge, Daniel J. Wilson, R. Craig MacLean

**Affiliations:** a Department of Zoology, University of Oxford, Oxford, UK; b Big Data Institute, Nuffield Department of Population Health, University of Oxford, Oxford, UK; University of Pittsburgh

**Keywords:** *Staphylococcus aureus*, ST239, MRSA, Antibiotic resistance, Evolution, Fitness, Genomics, horizontal gene transfer, Fitness cost

## Abstract

Selection plays a key role in the spread of antibiotic resistance, but the evolutionary drivers of clinically important resistant strains remain poorly understood. Here, we use genomic analyses and competition experiments to study Staphylococcus aureus ST239, a prominent MRSA strain that is thought to have been formed by large-scale recombination between ST8 and ST30. Genomic analyses allowed us to refine the hybrid model for the origin of ST239 and to date the origin of ST239 to 1920 to 1945, which predates the clinical introduction of methicillin in 1959. Although purifying selection has dominated the evolution of ST239, parallel evolution has occurred in genes involved in antibiotic resistance and virulence, suggesting that ST239 has evolved toward an increasingly pathogenic lifestyle. Crucially, ST239 isolates have low competitive fitness relative to both ST8 and ST30 isolates, supporting the idea that fitness costs have driven the demise of this once-dominant pathogen strain.

## INTRODUCTION

Antimicrobial resistance (AMR) in pathogenic bacteria has created a health care crisis by increasing the costs and mortality rates associated with bacterial infections. In many important pathogens, the rise of resistance has been driven by the epidemic spread of a small number of very successful clones, often those that have successfully acquired a range of resistance genes by horizontal gene transfer. One important challenge at the moment is to understand the evolutionary processes that give rise to these AMR superbugs (1), such as Staphylococcus aureus sequence type ST22 (2), Escherichia coli ST131 (3), and Klebsiella pneumoniae ST258 (4).

S. aureus is an important commensal pathogen that provides a clear illustration of the AMR crisis. Approximately one-third of humans show persistent asymptomatic carriage of S. aureus, mainly in the nares, but this bacterium is capable of causing serious invasive infections at a number of sites in the body (5). Antibiotic use has driven the epidemic spread of waves of resistant S. aureus strains; in particular, epidemic strains of methicillin-resistant S. aureus (EMRSAs) are now a global problem in health care settings and a growing problem in the community. For example, ST239 (a member of clonal complex 8 [CC8]) is a major EMRSA strain that has been the causative agent of multiple epidemics in health care settings around the globe. The first ST239 isolates were collected in the late 1970s in Australia (6). This was followed by the first EMRSA strain, also an ST239 and known as EMRSA-1, which emerged in the UK in 1981 (7). Between 1987 and 1988, more than 40% of MRSA isolates collected in England and Wales were ST239 EMRSA-1 (8), and ST239 became highly prevalent around the globe. By the mid-2000s, ST239 was the predominant MRSA strain in Asia, causing up to 90% of hospital-acquired MRSA within a region accounting for >60% of the world’s population (9). More recently, reports from around the globe show that levels of ST239 MRSA have been decreasing dramatically, but the causes of this decline remain unclear (10–12).

Robinson and Enright proposed that ST239 was formed as a result of a large-scale chromosomal replacement event in which an ST8 clone acquired ∼550 kb from an ST30 clone by recombination (13). Interestingly, five of the six known other cases of large-scale chromosomal replacement in S. aureus overlap to some extent with the acquired region of ST239 (13–16), suggesting that this region may be a recombination hot spot (17). Large-scale chromosomal replacements have also been detected in a number of important AMR pathogens other than S. aureus, including K. pneumoniae ST258, Campylobacter coli ST1150, and Streptococcus agalactiae (18–20). In ST239, this acquired region also contains a large (60-kb) SCC*mec*-III element that confers resistance to a broad spectrum of antibiotics and heavy metals, and ST239 is thought to have spread in health care settings around the world as a result of the selective advantage of the resistance provided by this acquired element (21).

Although subsequent studies have provided good support for the Robinson and Enright model, important questions regarding the origin of ST239 remain unresolved (22–25). All of the 2,979 ST239 genomes in the Staphopia database (26) that are associated with a typeable SCC*mec* element carry a type III SCC*mec* element, suggesting that this element was acquired early in the evolutionary history of ST239. However, the SCC*mec*-III element has not been identified in any ST30 genomes, including all 1,896 ST30 genomes in the Staphopia database. The absence of SCC*mec*-III in ST30 suggests that the true ancestor of ST239 may be a closely related lineage that has not been considered in previous studies of ST239. Second, the origins of ST239 remain unclear. It is often assumed that antibiotic-resistant pathogens evolve in response to treatment; in this case, it has been argued that the introduction of methicillin into clinical practice in the 1960s drove the evolution of ST239. However, recent work has shown that some other MRSA STs from CC8 predated the clinical introduction of methicillin (27), raising the possibility that ST239 may also have a deeper evolutionary history.

Finally, it is unclear why the prevalence of ST239 has recently declined. Acquiring DNA is usually associated with fitness costs (28–30), and many studies have shown fitness costs associated with acquired resistance genes (31–33), including large SCC*mec* elements (34, 35). Given these costs, it is possible that the recombination event(s) that gave rise to ST239 created a low-fitness “hybrid” pathogen, although this has not previously been investigated in detail.

Here, we use a combination of computational and experimental techniques to investigate the underlying evolutionary processes that have driven the rise of ST239. First, we assemble a diverse collection of ST239 genome sequences to reconstruct the evolutionary history of this lineage and to test the Robinson and Enright model. We then use competition assays to test the hypothesis that ST239 has low fitness and to explore the link between AMR and fitness. Finally, we use population genetic approaches to understand how selection has operated in ST239 and to identify key genes involved in adaptive evolution in this lineage.

## RESULTS AND DISCUSSION

### Reconstructing the origin of ST239.

The evolutionary drivers that contributed to the global emergence of the epidemic multidrug-resistant MRSA strain ST239 are not well understood; however, it has been suggested that the introduction of methicillin into clinics in the early 1960s may have been a contributing factor (36). To reconstruct the evolutionary history of ST239, we assembled a collection of 96 ST239 high-quality genomes from isolates that were collected from diverse geographic locations and time points, deliberately avoiding overrepresenting isolates from intensively sampled ST239 outbreaks (see, for example, references 37 and 22) (see Table S1A in the supplemental material).

10.1128/mBio.02168-21.5 TABLE S1 (A) Metadata for the ST239 sequences used in this study. (B) Metadata for the ST8 sequences used in this study. (C) Metadata for the ST30 sequences used in this study. (D) Metadata for the experimental sequences used in this study. The reference genomes for the ST239, ST8, and ST30 isolates were NCBI accession numbers FN433596, CP007690, and NC002952, respectively. Download Table S1, PDF file, 0.5 MB. Copyright © 2021 Gill et al. 2021 Gill et al. https://creativecommons.org/licenses/by/4.0/ This content is distributed under the terms of the Creative Commons Attribution 4.0 International license.

**FIG 1:**
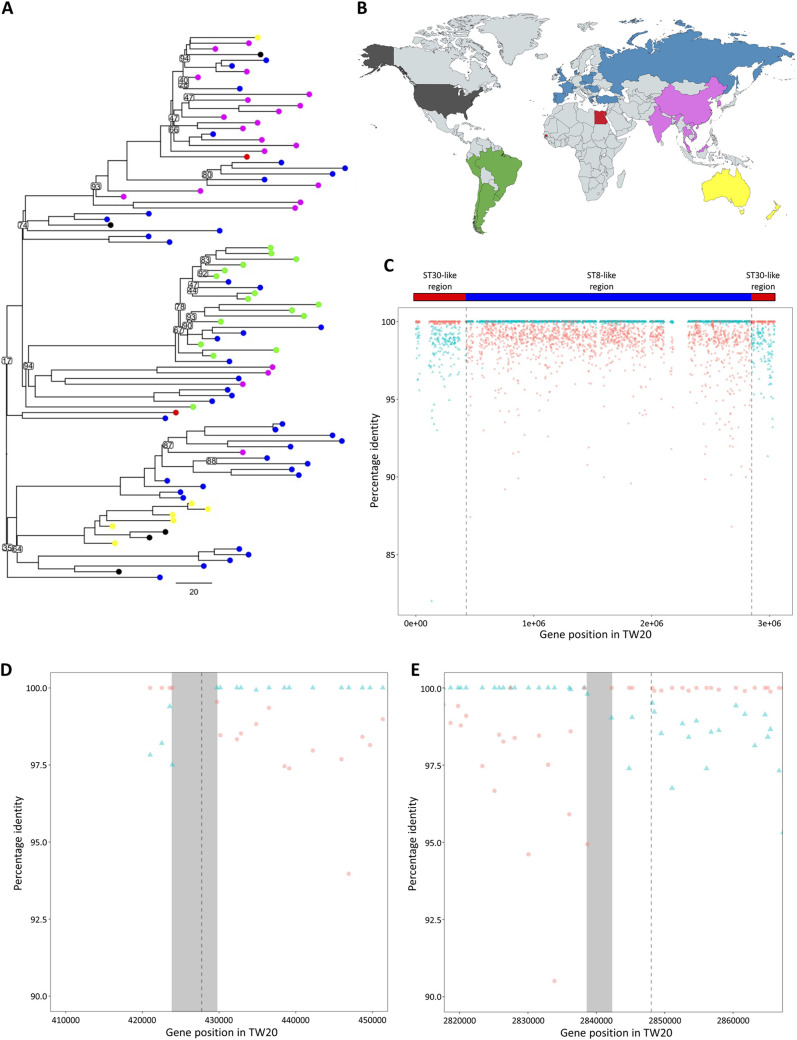
(A) Outgroup-rooted maximum-likelihood phylogeny of the 96 ST239 genomes. Branch lengths are shown in SNPs/Mb, and bootstrap support values below 95 are shown for branches. Isolates are color coded according to geographical origin, as displayed in panel B. Colors: blue, Europe; pink, Asia; yellow, Oceania; green, South America; black, North America; red, Africa. (C) BLAST DNA percentage identity of the ST8 (blue triangle) and ST30 (red circle) consensus core gene sequences compared to the ST239 consensus core gene sequences (consensus sequences were formed from 96 ST239 genomes, 111 ST8 genomes, and 57 ST30 genomes, for 1,980 core genes that were shared between all 264 genomes). In the ST239 genome, the acquired region spans the origin of replication and hence is split between the beginning and the end of the linearized genome. The hybrid boundaries estimated by Castillo-Ramirez et al. (24, 42) are highlighted with vertical dashed gray lines. (D) Closeup of the first boundary of the large chromosomal replacement event. (E) Closeup of the second boundary of the large chromosomal replacement event. The hybridization boundaries estimated by Castillo-Ramirez et al. (24, 42) are highlighted with vertical dashed gray lines, and the hybridization boundary ranges used in this study are highlighted by a gray rectangle. Each data point represents a single gene. The gene positions correspond to the genomic position within the ST239 reference genome.

We constructed a pangenome of 3,337 ST239 genes, of which 1,889 were present in 99 to 100% of all strains, resulting in a core genome length of 1,754,805 bp, representing a greater number of ST239 isolates from varied locations and dates than in previous evolutionary studies of ST239 (38–40). After we identified and excluded sites involved in recombination (41), 3,696 core variant sites remained, from which we reconstructed a phylogeny by maximum likelihood (Fig. 1A). The phylogeny had strong bootstrap support, and the long branch lengths were suggestive of a diverse set of ST239 isolates (38, 42). The clustering of isolates into geographically distinct clades is consistent with the population structure observed by Harris in 2010 and by Castillo-Ramirez in 2012, who identified strong geographical clustering of ST239 sequences on continental, national, and city scales (38, 42). In the present study, isolates from Oceania, Asia, and South America formed distinct clades, with rare exceptions. In contrast, North American and European isolates were dispersed throughout the tree.

We estimated a time to the most recent common ancestor (MRCA) of these ST239 sequences by fitting four evolutionary models using BEAST (see Table S2A) (43). The time to MRCA was consistent between all four models, with no significant difference between the models identified through Bayes Factor analysis. Therefore, the time to MRCA from the simplest model (strict molecular clock, constant population size) is recorded here, as 1940.1 (95% highest posterior density [HPD] intervals: 1934.7 to 1945.5). Similar results were obtained using BactDating (see Table S2B). Although there is some uncertainty in these estimates, these models predict that the origin of ST239 predated the clinical introduction of methicillin in 1959 (44) by more than 10 years.

10.1128/mBio.02168-21.6 TABLE S2 (A) BEAST model estimates of time to the MRCA of ST239. (B) BactDating model estimates of time to the MRCA of ST239. Download Table S2, PDF file, 0.1 MB. Copyright © 2021 Gill et al. 2021 Gill et al. https://creativecommons.org/licenses/by/4.0/ This content is distributed under the terms of the Creative Commons Attribution 4.0 International license.

### Identifying the donor of the acquired region of the ST239 genome.

Robinson and Enright initially proposed that ST30 was the closest known ancestor of the ST239 acquired-region (13), and they were able to identify potential boundaries of the recombination event that gave rise to ST239 (13). However, their conclusions were based on the partial sequencing of a small number of genes from representative isolates. To test the Robinson-Enright model using a large collection of genomes, we extracted the core genes shared by >99% of the isolates from the ST239 collection (*n *=* *96), combined with additional collections of diverse ST8 (*n *=* *111) and ST30 (*n *=* *57) genomes. A total of 1,980 core genes were identified that were shared between >99% of all 264 genomes. Within each ST (ST239, ST8, and ST30), we generated a consensus sequence for each of these genes, and calculated the percentage similarity between each ST239 gene and its homolog in either ST30 or ST8 (Fig. 1C).

There was a clear distinction between regions of the ST239 genome that were closely related to ST8 and ST30, allowing us to clearly differentiate between the backbone and acquired regions of the ST239 genome (Fig. 1D and E). This was consistent with Holden et al., who compared an ST239 genome sequence with a CC30 complete genome sequence and found that the acquired region was more closely related to CC30 by a shift of roughly 1% in the DNA percent identity compared to the backbone region (22).

**FIG 2:**
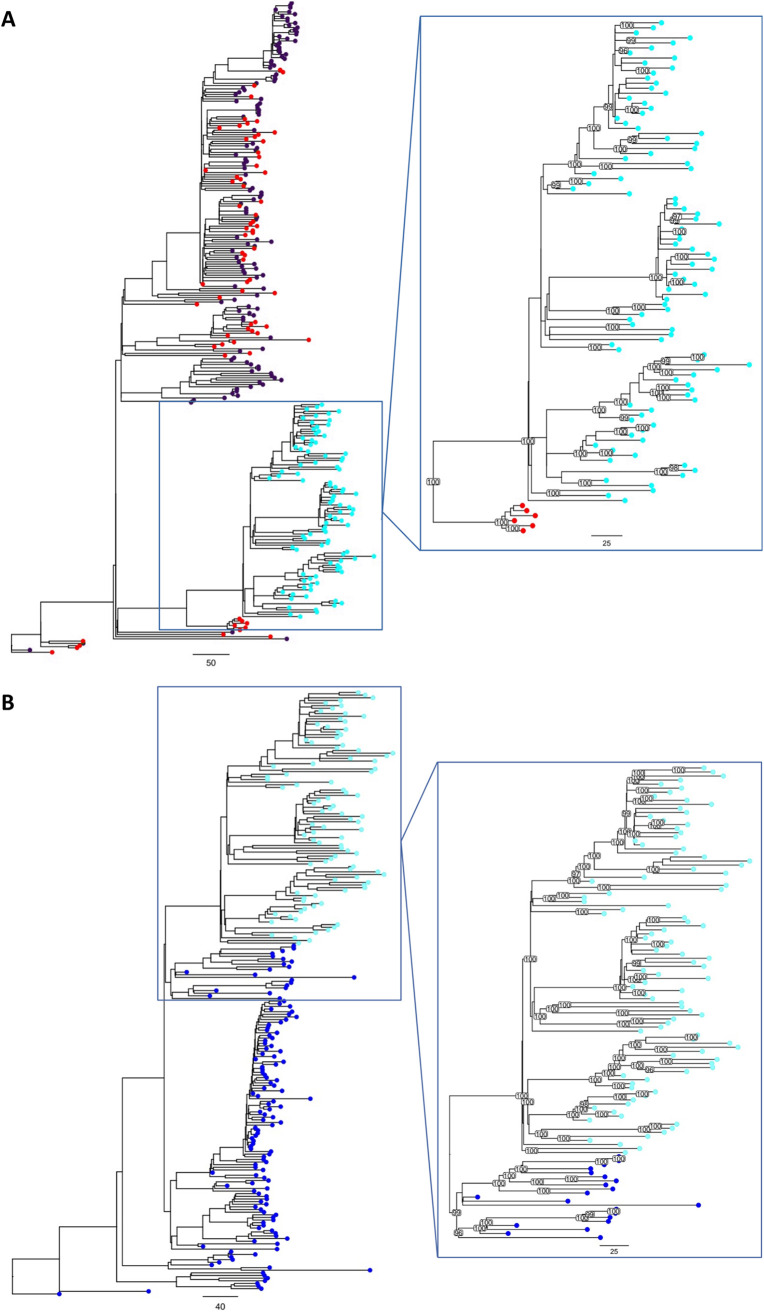
(A) Outgroup-rooted maximum-likelihood phylogeny of the acquired region of the ST239 genomes and the most closely related non-ST239-like isolates from BIGSI analysis. Branch lengths are shown in SNPs/Mb. ST239 isolates are in cyan, ST30 isolates are in red, and other STs are in purple. The zoom panel contains the ST239 clade and the most closely related non-ST239 isolates, with bootstrap support values >95 shown for branches. (B) Outgroup-rooted maximum-likelihood phylogeny of the backbone region of the ST239 genomes and the most closely related non-ST239-like isolates from BIGSI analysis. Branch lengths are shown in SNPs/Mb. ST239 isolates are in cyan, and ST8 isolates are in blue. The zoom panel contains the ST239 clade and the most closely related non-ST239 isolates, with bootstrap support values >95 shown for branches.

Although the acquired region of the ST239 genome is similar to ST30, it is possible that the true ancestor of this region was a closely related lineage of S. aureus that was not considered in previous analyses of ST239. For example, all of the 2,979 ST239 isolates in the Staphopia database that are associated with a typeable SCC*mec* element carry a type III SCC*mec* element (see Table S3), but this element has not been identified in any of the 1,896 ST30 genomes in the Staphopia database (26), suggesting that ST30 may be a sister group, rather than the true ancestor of the acquired region. To systematically search for the ancestor of the acquired region, we used BIGSI to screen 447,833 bacterial and viral raw-read and assembled genomes for short sequences that match those found in the acquired region of the ST239 genome (45). Sequences from isolates that were closely related to the acquired region of the ST239 genome were mapped to the acquired region of the ST239 reference genome (TW20, NCBI accession number FN433596) and assembled into a phylogeny alongside the acquired region of the 96 ST239 genomes (Fig. 2A). Crucially, we found that all ST239 isolates share a recent common ancestor with five ST30 isolates dating from the 1950s and 1960s (and one ST30 isolate of unknown origin), and this branch is well supported by bootstrapping. These five ST30 isolates (see Table S4A) were all from the penicillin-resistant S. aureus phage type 80/81 clone, which caused serious health care-associated and community-associated infections worldwide and was largely eliminated in the 1960s as a result of the introduction of methicillin (46, 47).

10.1128/mBio.02168-21.7 TABLE S3 SCC*mec* prevalence in ST239 sequences in Staphopia. SCC*mec* elements with “some evidence” had some SCC*mec* gene sequences present (e.g., *ccrA* or *mecA*) but not enough to provide a definitive SCC*mec* type. Download Table S3, PDF file, 0.1 MB. Copyright © 2021 Gill et al. 2021 Gill et al. https://creativecommons.org/licenses/by/4.0/ This content is distributed under the terms of the Creative Commons Attribution 4.0 International license.

10.1128/mBio.02168-21.8 TABLE S4 (A) Accession numbers of the ST30 genomes that share the closest common ancestor with the acquired region of the ST239 genome. (B) Accession numbers of the ST8 genomes that share the closest common ancestor with the backbone region of the ST239 genome. Download Table S4, PDF file, 0.1 MB. Copyright © 2021 Gill et al. 2021 Gill et al. https://creativecommons.org/licenses/by/4.0/ This content is distributed under the terms of the Creative Commons Attribution 4.0 International license.

Interestingly, none of the phage type 80/81 genomes that are closely related to ST239 contain an SCC*mec* element. The ubiquity of SCC*mec*-III in ST239 genomes suggests that the SCC*mec*-III element was acquired by the ancestor of the acquired region of ST239 following the divergence of this lineage from the phage type 80/81 lineage. However, it is possible that the SCC*mec*-III element was secondarily acquired following the chromosomal replacement that gave rise to ST239. To test the secondary acquisition hypothesis, we estimated the time to MRCA of the SCC*mec*-III elements in the collection of ST239 isolates (see Fig. S1 and Table S2A). However, there was considerable uncertainty in this estimate due to the small size of the SCC*mec*-III element (60 kb) and the high rate of recombination in this region of the genome. Given, these limitations, this analysis had limited power to reject the null hypothesis that the SCC*mec*-III element was acquired as part of the initial chromosomal replacement event that gave rise to ST239.

10.1128/mBio.02168-21.1 FIG S1 Midpoint rooted maximum-likelihood phylogeny of the SCC*mec*-III region of ST239. Units in total number of SNPs. Download FIG S1, PDF file, 0.2 MB. Copyright © 2021 Gill et al. 2021 Gill et al. https://creativecommons.org/licenses/by/4.0/ This content is distributed under the terms of the Creative Commons Attribution 4.0 International license.

As a final test of the Robinson-Enright model, we used BIGSI to identify the closest known ancestor of the backbone region of the ST239 genome. Consistent with the Robinson and Enright model, this analysis identified ST8 as the closest ancestor of the ST239 backbone region (13). Specifically, the ancestor of ST239 was part of a diverse lineage of ST8 that has been isolated across multiple continents over the last 70 years (Fig. 2B; see also Table S4B).

Dating the MRCA of ST239 isolates places an upper bound on the origin of ST239, but it is possible that ST239 originated prior to the MRCA of contemporary isolates. To place a lower bound on the origin of ST239, we estimated times to the MRCA of the acquired and backbone regions of ST239 with their respective ST8 and ST30 ancestors. The MRCA of the acquired region and ST30 was estimated as 1900.3 to 1926.5 (95% HPD), and the MRCA of the backbone region and ST8 was estimated as 1924.5 to 1934.7 (95% HPD). The overlap of these two estimates is encouraging, and this analysis suggests that ST239 is unlikely to have originated prior to the 1920s.

### Recombination.

**FIG 3:**
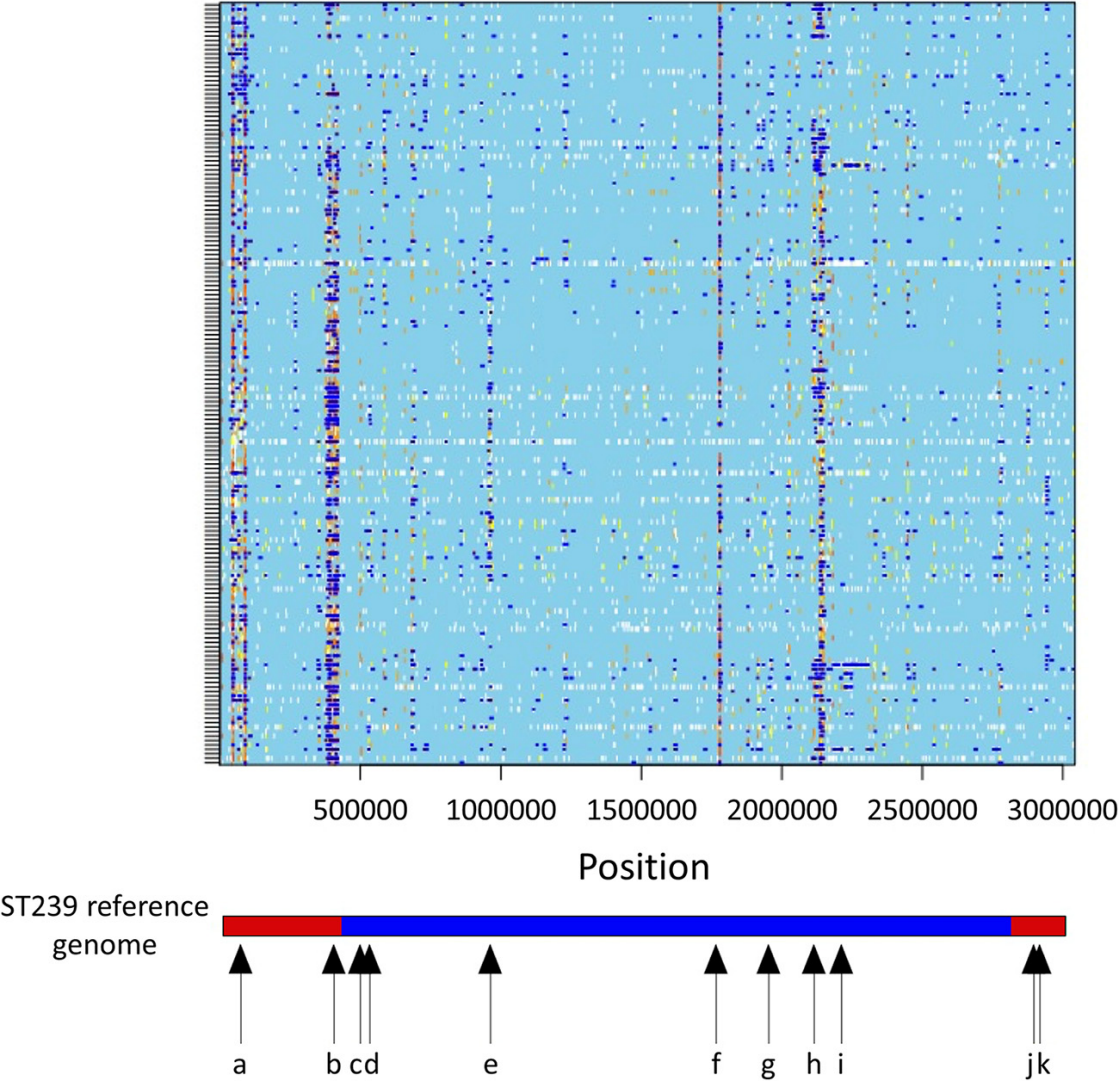
Recombination plot of the ST239 genomes compared to the ST239 TW20 reference genome. Dark blue regions represent potential sites of recombination. Sites that are nonpolymorphic are shown in light blue. Polymorphic sites are shown in a color indicating their level of homoplasy; white indicates no homoplasy, and the range from yellow to red indicates increasing degrees of homoplasy. The positions of known MGEs (a, MGE SCC*mec*-III; b, prophage ϕSa1 [TW20]; c, transposon Tn*5801*; d, genomic island υSaα; e, pathogenicity island SaPI1; f, transposon Tn*554*; g, genomic island υSaβ; h, prophage ϕSa3(TW20); i, prophage ϕSaβ-like(TW20); j, transposon Tn*55*2; k, ICE 6013) and the two inherited regions estimated by Holden et al. and Castillo-Ramirez et al. are highlighted along the ST239 reference genome beneath the plot (green, acquired region; magenta, backbone region).

Given that recombination created the ST239 lineage, it is possible that recombination has also played an important role in the subsequent evolution of ST239. To investigate this idea, we used ClonalFrameML to identify regions of recombination within the collection of 96 ST239 genomes (Fig. 3; see also Fig. S2). Ten isolates from the TW20-like clade were excluded from this analysis due to high sequence similarity to the reference sequence that all ST239s were mapped to.

10.1128/mBio.02168-21.2 FIG S2 Number of nodes and tips affected by recombination, as estimated by ClonalFrameML, at each position along the TW20 genome. Regions of high recombination are labeled 1, 2, 3, and 4. Download FIG S2, PDF file, 0.2 MB. Copyright © 2021 Gill et al. 2021 Gill et al. https://creativecommons.org/licenses/by/4.0/ This content is distributed under the terms of the Creative Commons Attribution 4.0 International license.

The ratio of rates of recombination and mutation (*R*/*θ*) was 0.41, and the ratio of effects of recombination and mutation (*r/m*) was 2.37. Therefore, recombination events occurred 2.5 times less often than mutations; however, because each recombination event introduced an average of 6.0 substitutions, recombination overall was responsible for 2.4 times more substitutions than mutations (48). In line with previous analyses, we found evidence of recombination hot spots in regions of the genome containing mobile genetic elements (24). Recombination was particularly frequent in the region surrounding the ϕSA1 prophage, suggesting that this element, which borders the acquired region, may have played a key role in the recombination event that gave rise to ST239. Notably, the SCC*mec*-III element is also a hot spot for recombination.

### Evolutionary consequences of large-scale chromosomal replacement.

To begin to understand the evolutionary consequences of large-scale chromosomal replacement, we calculated the evolutionary rate of the backbone and acquired regions of the ST239 genome using BEAST (see Table S2A) (43). After removing recombination and the SCC*mec* element, the overall genomic substitution rate was estimated as 1.205 × 10^−6^ single nucleotide polymorphisms (SNPs)/site/year (1.13 × 10^−6^ to 1.28 × 10^−6^ SNPs/site/year; 95% HPD), which is similar to the substitution rates of other EMRSAs (2, 49–51). Interestingly, the substitution rate of the acquired region, 1.515 × 10^−6^ (1.32 × 10^−6^ to 1.71 × 10^−6^ SNPs/site/year; 95% HPD), was 2 to 51% more rapid than the backbone, 1.21 (1.13 × 10^−6^ to 1.29 × 10^−6^ SNPs/site/year; 95% HPD). On the one hand, the rapid evolution of the acquired region could be a signature of rapid adaptive evolution, perhaps to overcome the costs associated with horizontal gene transfer. Alternatively, it is possible that the acquired region has evolved at a high rate due to weak selective constraints in this region of the genome.

To understand how selection has acted in the two genetic regions of the ST239 genome, we used the McDonald-Kreitman test to compare patterns of polymorphism and divergence in the backbone and acquired regions of the genome (52). The McDonald-Kreitman net neutrality index (N) indicates whether selection is overall purifying (N > 1) or positive (N < 1) (53).

In this analysis, we compared the acquired region of the 96 ST239 genomes with the homologous region in the previously defined collection of 57 ST30 genomes. In addition, we compared the backbone region of the 96 ST239 genomes with the homologous region in the previously defined collection of 111 ST8 genomes. Only “core” genes that were shared by more than 99% of all ST239, ST8, and ST30 genomes in the collections were included. The divergence between ST239 and ST30 in the acquired region (0.326 substitutions/Mb) was much greater than the divergence between ST239 and ST8 in the backbone region (0.188 substitutions/Mb), reflecting the fact that ST239 is more closely related to ST8 than ST30 in the respective regions. Divergence between STs at nonsynonymous sites was low relative to levels of within-ST polymorphism, demonstrating an overall trend toward purifying selection in ST239 (Table 1; N >1). However, we did not find any difference in the net neutrality index between the backbone (N =* *2.06; 95% confidence interval [CI] = 0.92 to 2.51) and the acquired region (N* *=* *1.52 [95% CI = 1.34 to 3.19]).

**TABLE 1:** McDonald-Kreitman net neutrality index for the two genetic regions of ST239^*a*^

Region	Comparison	D_n_	D_s_	D_n_/D_s_	P_n_	P_s_	P_n_/P_s_	N	Selection
Acquired	ST239/ST30	40	32	1.25	1,406	740	1.90	1.52	Negative
Backbone	ST239/ST8	42	50	0.84	6,080	3,508	1.73	2.06	Negative

a D_n_, fixed nonsynonymous substitutions; D_s_, fixed synonymous substitutions; P_n_, nonsynonymous polymorphisms; P_s_, synonymous polymorphisms; N, McDonald-Kreitman net neutrality index.

One weakness of this approach in this context is that the backbone and acquired regions of the ST239 genome have to be compared to different outgroups. Given that signatures of purifying selection should become stronger over time, it could be argued that the McDonald-Kreitman test is biased toward detecting purifying selection in the acquired region of the genome, which was compared to a more divergent outgroup. To further test the idea that selection on the acquired region has been weak, we calculated the ratio of GC→AT to AT→GC substitutions in each region of the ST239 genome (Table 2). Spontaneous mutation in S. aureus is biased toward GC→AT transitions, and regions of the genome that are subject to weak selective constraints are therefore expected to have a high ratio of GC→AT to AT→GC substitutions (54). In this case, the GC→AT/AT→GC ratio in the acquired region was significantly greater than that of the backbone region (Fisher exact test, odds ratio = 1.15, *P = *0.0303). This analysis, which uses data on substitutions that have occurred during the diversification of ST239, provides evidence of relaxed selective constraints on the acquired region of the ST239 genome.

**TABLE 2:** Total number of GC→AT and AT→GC substitutions in the acquired and backbone regions of the ST239 genome

Substitution	Total no. of substitutions	
	Acquired region	Backbone region
GC→AT	795	2,710
AT→GC	474	1,864

### Fitness costs of chromosomal replacement.

To understand the fitness effects of chromosomal replacement more directly, we measured the competitive ability of a collection of S. aureus isolates *in vitro* (see Fig. S3). Our collection of isolates included divergent isolates of ST239 (*n *=* *4), ST8 (*n *=* *6), and ST30 (*n *=* *5) that were deliberately chosen to avoid, including clonal isolates within STs (see Table S1D). If chromosomal replacement is costly, then we would expect ST239 isolates to have low competitive ability relative to ST8. The additional ST30 isolates provided a useful reference point to compare fitness values. Isolates were directly competed against each other in three different culture media; tryptic soy broth (TSB), brain-heart infusion (BHI) broth, and porcine serum (PS). These media impose different stresses that mimic some of the challenges encountered by S. aureus in clinical environments.

10.1128/mBio.02168-21.3 FIG S3 (A) Schematic of unique variant site identification protocol. (B) Schematic of competition experimental protocol. (C) Schematic of protocol to estimate competitive ability. Download FIG S3, PDF file, 0.4 MB. Copyright © 2021 Gill et al. 2021 Gill et al. https://creativecommons.org/licenses/by/4.0/ This content is distributed under the terms of the Creative Commons Attribution 4.0 International license.

**FIG 4:**
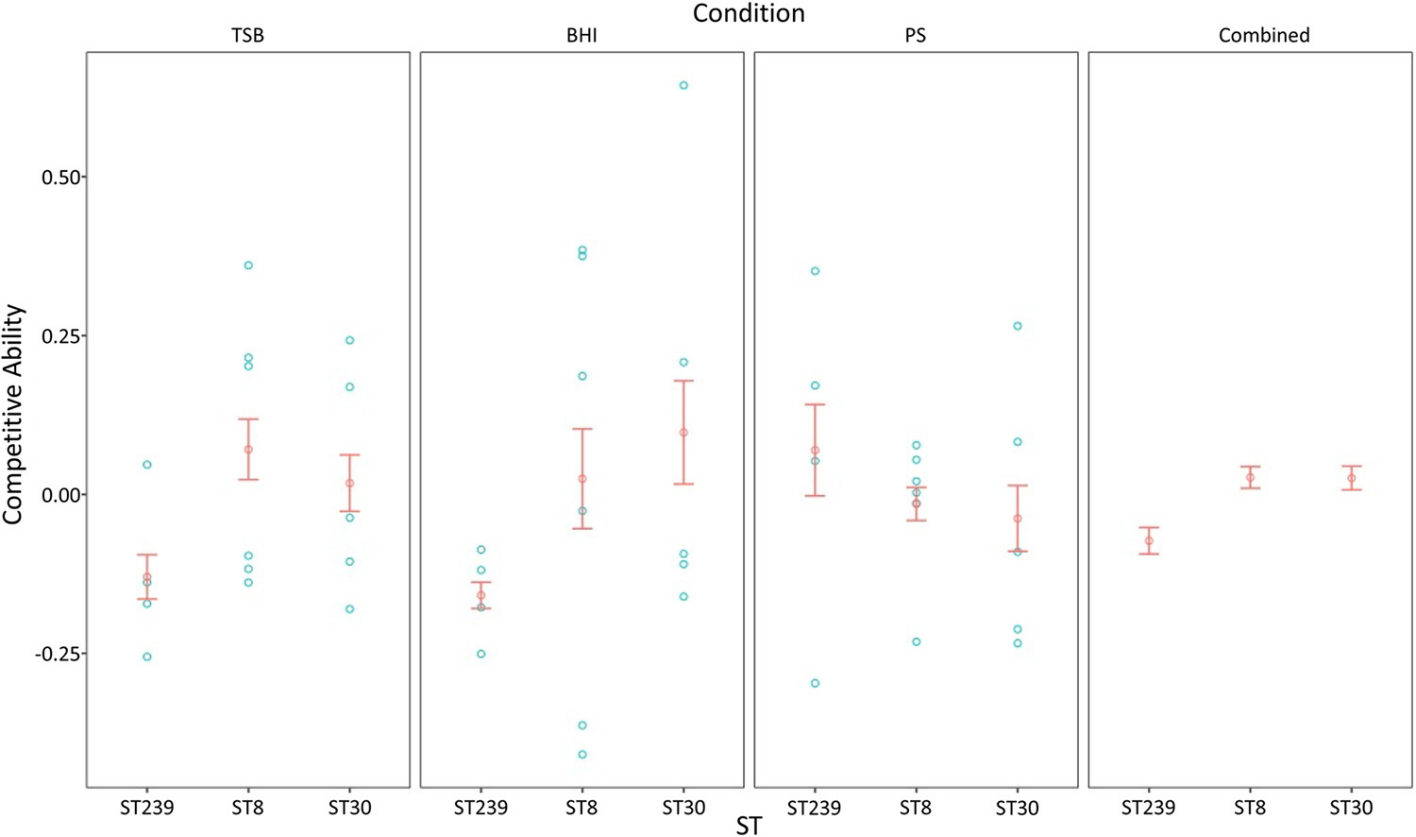
Competitive ability. Plotted points show the competitive ability of S. aureus isolates (blue circles) and the mean competitive ability of each ST (red circles; ± standard errors of the mean [SEM]; *n *= 4 to 6). Competitive ability was measured in all-versus-all coculture experiments. Competitive ability was measured in triplicate assays, and the errors for individual isolates were minor (SE = 0.045 to 0.001). ST239 isolates have reduced competitive ability relative to ST8 and ST30 in BHI and TSB, but not in PS, as judged by a *post hoc* Tukey test (*P* < 0.05). The final panel shows the overall effect of ST on competitive ability (± the SE) across all three media.

We deep sequenced (142× to 211× depth) each competition mixture before and after growth and estimated changes in the relative abundance of each isolate by quantifying the relative abundance of isolate-specific SNPs during competition. This assay produced highly reproducible measures of competitive ability for individual combinations of isolate and condition (median coefficient of variation = 16%; interquartile range = 10 to 40%; *n* = 35). The competitive ability varied between isolates, and we found a strong statistical interaction between ST and media, which reflects the fact that the average competitive ability of the three STs varied across media (Fig. 4, the first three panels, and Table 3; see also Table S5A). For example, we did not find any evidence of low fitness associated with ST239 in porcine serum. In spite of this variation in fitness, we found a significant difference in competitive ability between STs. Crucially, we found that ST239 had lower competitive ability than both ST8 and ST30 (Fig. 4, fourth panel; *P* < 0.05 [*post hoc* Tukey test]). The low fitness of ST239 relative to the ST8 is consistent with the idea that chromosomal replacement carries a long-term fitness cost. This hypothesis is further supported by the high fitness of ST30, which suggests that the difference in fitness between ST239 and ST8 reflects a low fitness of ST239 rather than a high fitness of ST8.

**TABLE 3:** Reduced ANOVA TABLE of significant competitive ability effects

Effect	df	Sum of squares	Mean square	F-value	*P*
ST	2	0.260	0.130	8.33	0.0004
Media × ST	4	0.565	0.141	9.05	<0.0001
Isolate[ST]	12	4.24	0.354	22.6	<0.0001
Error	116	1.81	0.0156		

10.1128/mBio.02168-21.9 TABLE S5 (A) Reduced ANOVA table of significant competitive ability effects, with the medium effect removed. (B) Reduced ANOVA table of significant growth rate effects, with the medium effect removed. Download Table S5, PDF file, 0.1 MB. Copyright © 2021 Gill et al. 2021 Gill et al. https://creativecommons.org/licenses/by/4.0/ This content is distributed under the terms of the Creative Commons Attribution 4.0 International license.

As a complementary approach to measure fitness, we also measured the growth rate of the individual ST239, ST8, and ST30 isolates in TSB, BHI and PS (see Fig. S4). There was significant variation in growth rate between isolates, media, and ST, showing that this trait is highly variable (see Table S5B). Crucially, we found that ST239 isolates had significantly lower growth rate than ST8 isolates, providing further evidence of costs associated with chromosomal replacement.

10.1128/mBio.02168-21.4 FIG S4 Growth rates (± the standard error [SE]; *n *=* *9) of ST239, ST8, and ST30 isolates in TSB (A), BHI (B), and PS (C). (D) Mean growth rate (± the SE) of each ST across all three media. Both ST239 and ST30 have reduced growth rate relative to ST8 (ANOVA; *P* < 0.05). Cryostocks of the 15 isolates were streaked on TSA, followed by incubation at 37°C for 24 h. Single colonies were incubated for 24 h in 3 mL of TSB at 37°C with 225 rpm shaking. The, 1 mL of each culture was pelleted and washed three times in PBS, followed by dilution 50× in either TSB, BHI, or PS. Next, 100 μl of diluted culture was added to each well of a 96 well plate, in triplicate, followed by incubation at 37°C with 225-rpm shaking. The OD_595_ was measured every 20 min over 24 h or until the growth rate had reached a plateau. This was repeated five times for each isolate. Growth curves were generated and analyzed with Growthcurver (88) to calculate an exponential growth rate for each culture. Download FIG S4, PDF file, 0.2 MB. Copyright © 2021 Gill et al. 2021 Gill et al. https://creativecommons.org/licenses/by/4.0/ This content is distributed under the terms of the Creative Commons Attribution 4.0 International license.

### Parallel evolution.

A recurring theme of studies of microbial evolution is that genes that are under strong positive selection evolve in parallel (55–57). To better understand the selective pressures that have shaped the evolution of ST239, we compared the distribution of observed mutations per gene with a neutral model derived from the Poisson distribution, in which mutations are randomly distributed across genes. This analysis was carried out independently for the acquired and backbone regions of the ST239 genome to take into account the differences in substitution rate between these genomic regions. Only the 1,980 “core” genes that were shared between the previously defined ST239, ST30, and ST8 genome collections were included.

The number of mutations per gene differed from the Poisson expectation in both the acquired region (χ^2^ = 19.158, *P = *0.0014) and the backbone region (χ^2^ = 177.8, *P* < 0.0001), showing that substitutions are nonrandomly distributed across the ST239 genome. A subset of genes that show more evidence of parallel evolution than expected due to chance alone were defined as those that had 9 or more substitutions per gene. Our justification for this cutoff is that the Poisson distribution predicts that 1 or 2 genes in each region of the genome should have acquired 9 mutations or more due to chance alone, given an average of 2.53 substitutions per gene in the backbone region and 2.97 substitutions per gene in the acquired region. The proportion of genes showing evidence of parallel evolution did not differ between the backbone (20/1,659 = 1.21% genes) and acquired regions (6/316 = 1.90% genes), indicating that genes under positive selection are evenly distributed across the ST239 genome (chi-squared test, χ^2^ = 0.9818, *P = *0.3218). However, of the genes showing evidence of parallel evolution, those in the acquired region had a much larger proportion of substitutions (*N *=* *154 substitutions, 15.75%) than the backbone region (*n *=* *284 substitutions; 6.61%). The acquired region includes the *spa* gene (*n *=* *62 substitutions), which is potentially prone to sequencing errors due to the large number of repeats within this gene. Even after we excluded this gene, the remaining genes showing evidence of parallel evolution in the acquired region are significantly enriched for substitutions compared to those in the backbone (chi-squared test, χ^2^ = 13.111, *P = *0.000294). The high rate of substitutions in these genes suggests that the acquired region has been a hotspot for adaptive evolution in the ST239 genome. Note that this analysis is robust to the overall elevated substitution rate of the acquired region because it is based on a subset of genes that show a high rate of substitution compared to other genes in the region.

**FIG 5:**
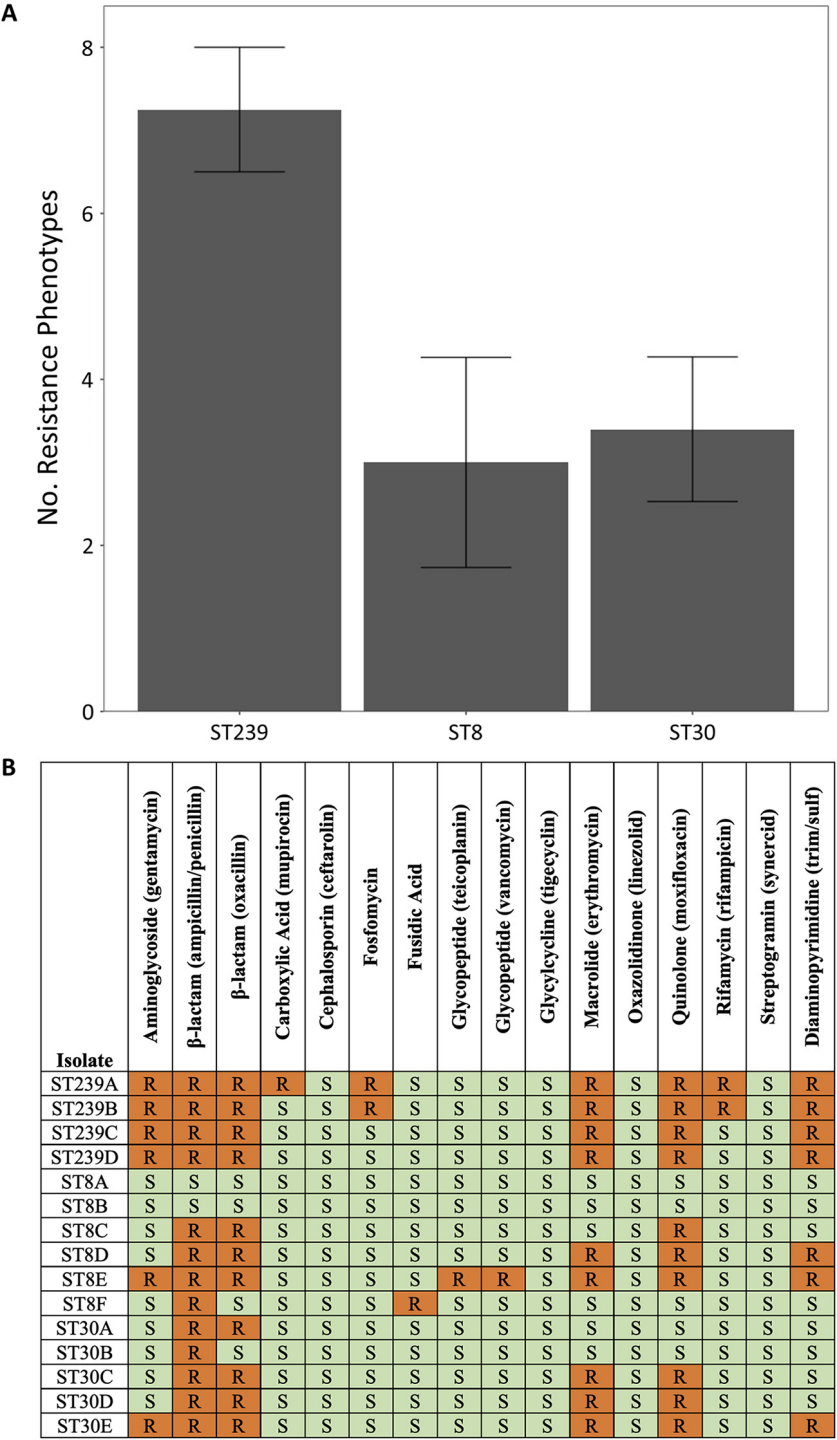
(A) Mean (± the SEM; *n *=* *4 to 6) numbers of AMR phenotypes for ST239, ST8, and ST30 isolates. (B) Heat maps of AMR phenotypes for ST239, ST8, and ST30 isolates. Orange (R), resistant; green (S), susceptible.

Interestingly, many (*n *=* *7) of the genes that show evidence of parallel evolution (see Table S6) are involved in resistance to antibiotics, including vancomycin/daptomycin (*walK*), fluoroquinolones (*grlA*), β-lactams (*ponA* and *mprF*), and rifampicin (*rpoB*). To test for elevated resistance at phenotypic level, we measured the resistance of our isolates to a broad panel of antibiotics that have activity against S. aureus. ST239 isolates were resistant to a greater number of antibiotics (Fig. 5; mean = 7.25; standard error [SE] = 0.75) than either ST30 or ST8 (one-way analysis of variance [ANOVA], followed by Hsu’s test F_2,12_ = 5.63; *P = *0.0187), highlighting the high levels of AMR associated with this lineage. Given that antibiotic resistance tends to be costly, we tested for a tradeoff between resistance (measured as the number of resistance phenotypes) and the competitive ability at the level of individual isolates. We did not find tradeoffs between AMR and fitness in any culture medium (*n* = 3 conditions, *P* > 0.3), nor did we find a tradeoff between AMR and the overall average competitive ability of each isolate across all three conditions (F_1,13_ = 1.33; *P* = 0.27; r^2^ = 0.09). This analysis suggests that although ST239 has high resistance and low fitness, the low fitness of ST239 cannot simply be explained by the high number of resistant determinants found in this strain.

10.1128/mBio.02168-21.10 TABLE S6 Candidate genes in ST239 that show potential evidence of parallel evolution. Gene names and functions were identified in AureoWiki (89). Download Table S6, PDF file, 0.2 MB. Copyright © 2021 Gill et al. 2021 Gill et al. https://creativecommons.org/licenses/by/4.0/ This content is distributed under the terms of the Creative Commons Attribution 4.0 International license.

### Conclusion.

In line with previous work, our results support the hypothesis that ST239 was produced by a large-scale chromosomal replacement event in which an ST8 clone acquired >600 kb of DNA from an ST30 clone (13, 22). We were able to refine this model by showing that the ancestor of the acquired region of the ST239 genome was most closely related to phage type 80/81 clones that were associated with the epidemic spread of penicillin resistance in the 1950s and 1960s (58). The most parsimonious explanation for the presence of the SCC*mec*-III in ST239 is that this element was acquired by the ST30 ancestor of ST239, following the divergence of this lineage from phage type 80/81. However, our analysis had limited power to detect secondary acquisition of SCC*mec*-III. We estimate that ST239 originated between the 1920s and 1945, in contrast to a previous study (25), but providing further support to other evidence that MRSA predated the clinical introduction of methicillin in 1959 (44). SCC*mec*-III provides resistance to first generation antibiotics that were used prior to the introduction of methicillin, such as tetracycline and erythromycin, and heavy metals, such as cadmium and mercury, that are used in disinfectants and biocides in health care settings (59, 60), suggesting that these resistance phenotypes may have provided ST239 with a selective advantage prior to the introduction of methicillin.

Fitness costs of laboratory-evolved antibiotic resistance have been demonstrated in many studies (31, 61, 62). Although resistance tends to carry a cost, it is important to emphasize that this is an overall statistical trend, and it is clear that fitness effects of resistance are shaped by bacterial genetic background and culture conditions (63–66). The cost of resistance in clinical pathogen populations have received less attention (66–68), making it difficult to predict how much variation in fitness to expect between resistant and sensitive strains. However, the variability in the fitness effect of resistance mutations across strains and conditions suggests that the fitness of clinical isolates should be variable. To take this variability into account, we measured fitness of multiple isolates of each ST across a range of culture conditions. We found extensive variation in competitive fitness between S. aureus isolates and culture conditions. In spite of this variation, we found a clear overall trend toward low fitness in ST239 relative to ST8, providing good evidence of a fitness cost associated with the evolution of elevated antibiotic resistance. This hypothesis is further supported by epidemiological evidence. ST8 is primarily found in the community, where antimicrobial use is low, whereas ST239 has mainly been restricted to health care settings where antimicrobial use is high, suggesting that the low fitness of this ST has restricted the spread of ST239 into the community (22, 69, 70). The acquisition of large SCC*mec* elements tends to generate a fitness cost, suggesting that the SCC*mec*-III element (which is the largest known SCC*mec* element) contributes to the low fitness of ST239 (see also reference 67). Although we found some evidence that the acquired region of the ST239 genome is subject to relaxed selective constraints, the evolution of this region is dominated by purifying selection, suggesting that the chromosomal replacement may have had costs beyond those associated with the acquisition of SCC*mec*-III.

Although the evolution of ST239 has been dominated by purifying selection, we found evidence of positive selection in genes that are implicated in antibiotic resistance, virulence and metabolism. Notably, many of the genes that show clear hallmarks of lineage-specific positive selection in the ST239 genome are associated with resistance to antibiotics that have been used to treat MRSA infections, such as ciprofloxacin (*grlA*), vancomycin (*walK*), and rifampicin (*rpoB*). These patterns of parallel evolution suggest that the ST239 has evolved to increase antibiotic resistance and virulence rather than to overcome the costs associated with chromosomal replacement and SCC*mec*-III acquisition.

Bacterial recombination is typically associated with the exchange of short DNA sequences between closely related strains or species (71), and the large-scale chromosomal replacements that have been detected in pathogenic bacteria are conspicuous exceptions to this overall trend (18–20). Our results support the idea that ST239 is a “hopeful monster” that has declined in prevalence due to fitness costs of chromosomal replacement, and an important goal for future work will be to understand the fitness consequences of chromosomal replacement in other dominant hybrid pathogens.

## MATERIALS AND METHODS

### Genome data retrieval and processing.

Sequences with an MLST profile corresponding to ST239 were identified within the Staphopia database (26). The isolation date and geographic location were extracted from the metadata files. Additional ST239 sequences with corresponding isolation date and location metadata were identified from the NCBI database using MegaBLAST (72) and through a literature search. A total of 96 ST239 sequences were selected and downloaded from EMBL-EBI (see Table S1A). Similarly, a total of 57 ST30 and 111 ST8 genomes were identified and downloaded from EMBL-EBI (see Table S1B and C).

Where the sequences were downloaded as assemblies or complete genomes, raw sequence reads were simulated with dwgsim v0.1.11 (73). Illumina reads were trimmed using Trimmomatic (74), and bwa v0.7.15 and SAMtools v1.3.1 (75) were used to map the ST239 sequences to the ST239 reference sequence (accession number FN433596). ST30 sequences were mapped to both the ST239 and ST30 reference sequences (accession number LN626917); similarly, the ST8 sequences were mapped to the ST239 and ST8 reference sequences (accession number CP007690).

The boundaries of the SCC*mec*-III element were identified in the ST239 reference genome using BLAST against the reference SCC*mec*-III element under accession number AB03767159. The boundaries were confirmed using SCC*mec*Finder (76).

The SCC*mec* type of all 2,979 ST239 sequences in the Staphopia database was predicted using the Staphopia API. The Staphopia database was also mined for all sequences predicted to contain SCC*mec*-III. The MLST of these sequences was identified using PubMLST (77). The MLST was confirmed using ARIBA v2.13.3 (78), and the SCC*mec* type was confirmed using SCC*mec*Finder.

### Construction of ST239 phylogeny.

RaxML v8.2.9 (79) was used to construct a maximum likelihood phylogeny from the collection of 96 ST239 genomes that had been mapped to the ST239 reference sequence, using a GTR model with gamma correction for among site rate variation, which was replicated for 100 bootstraps, with recombination masked using Gubbins (41). The ST8 reference sequence, mapped to the ST239 reference sequence, was used as an outgroup. Genes were annotated with Prokka v1.13 (80). Gene function for genes that could be annotated by Prokka was identified in UniProt, and MegaBLAST (71) was used to identify genes where no annotation was found with Prokka. The pangenome and core genome (genes shared by >99% of the ST239 isolates) was extracted using Roary v3.12.0 (81).

### Estimation of time to the MRCA of the ST239 collection.

The time to the MRCA for the collection of 96 ST239 genomes was initially estimated from the maximum likelihood phylogeny (with recombination masked using Gubbins [41]), using the BactDating R package linear regression function (82). Mixed-gamma, strict-gamma, and relaxed-gamma evolutionary models were run for 10,000,000 MCMC steps to estimate a time to the MRCA of the ST239 whole-genome sequences. The effective sample size (ESS) values for all parameters were greater than 100, indicating adequate sampling of the posterior distribution.

A total of 6,819 variant sites were extracted from the ST239 sequence alignments, after recombination was masked using Gubbins (39), using snp-sites v2.3.3 (83). Bayesian phylogenetic analysis was also carried out using BEAST version 1.10.4 (43), using the GTR nucleotide substitution model with all combinations of the strict and uncorrelated relaxed molecular clock models and constant and exponential growth models. The XML file was edited to reflect the number of unchanging sites in the original alignments. For each model, three independent MCMC chains with 300,000,000 steps were run and combined, with path sampling/stepping-stone sampling every 100 steps. In all cases, the Bayes Factor showed no significant difference in the likelihood of the different models, and therefore the estimated time to the MRCA from the simplest model (strict molecular clock, constant population size) was recorded. The burn-in was set at 10%, and runs were combined using LogCombiner, with a resample size of 10,000. The MRCA and evolutionary rates were estimated with 95% HPD intervals.

BEAST analysis was repeated for 95 ST239 SCC*mec*-III element sequences (*n = *54 variant sites), using the same clock and nucleotide substitution models as previous (one ST239 sequence was removed from the analysis due to low mapping quality of the SCC*mec*-III region).

### BLAST comparison of the ST239, ST30, and ST8 core genes.

A multiple sequence alignment was constructed from the 96 ST239, 57 ST30, and 111 ST8 genomes that had been mapped to the ST239 reference genome. The shared pan-genome was calculated (*n *=* *2,962 genes), and the core genes that were shared between >99% of all 264 genomes were extracted using Roary v3.12.0 (81) (*n *=* *1,980 core genes). EMBOSS (84) was used to generate ST-specific consensus sequences from the core genes of each ST (ST239, ST8, and ST30). For each gene in the ST239 consensus sequence, the percentage identities compared to the homologous gene in the ST30 and ST8 consensus sequences were calculated using MegaBLAST (71). The ST239 core genes were defined as “acquired” (i.e., from the ST30-like region) or “backbone” (i.e., from the ST8-like region) depending on their position in the genome and similarity to the ST30 and ST8 consensus sequences.

### Identifying the closest known ancestor of the acquired and backbone regions.

The consensus sequences of the 316 core genes from the acquired region of the 96 ST239 sequences were queried for similar sequences in BIGSI using a k-mer threshold of 99. This allowed for an average divergence of around 10 SNPs per gene. The most closely related sequences were identified by ST using the Staphopia API. The MLST was double checked using ARIBA, and 28 sequences were removed due to uncertainty in typing, which indicated contamination.

The consensus sequences of the 1,659 core genes from the backbone region of the 96 ST239 sequences were also queried for similar sequences in BIGSI using a k-mer threshold of 99, as well as Staphopia, as described above. This allowed for an average divergence of around 9 SNPs per gene.

Sequences that shared at least 99% k-mer identity with >200 of the ST239 core genes from the acquired region and shared two or fewer MLST alleles with ST239 were downloaded from the EBI database. Only a selection of the 12 most closely related ST30, ST36, and ST39 sequences were included. These 143 sequences were mapped to the ST239 TW20 reference sequence, as described above. Four sequences were removed due to poor mapping quality (>25% gaps). The acquired region was extracted (minus the SCC*mec* element) and combined into a multifasta alignment with the acquired regions from the 96 ST239 sequences and 57 ST30 sequences that were described previously.

Sequences that shared at least 99% k-mer identity with >1,400 of the ST239 core genes from the backbone region and were not previously identified as ST239-like were then downloaded from the EBI database. All 32 non-ST239-like sequences were identified using the Staphopia API as ST8. These 32 ST8 sequences were mapped to the ST239 TW20 reference sequence, as described above. The backbone region was extracted and combined into a multifasta alignment with the backbone regions from the 96 ST239 sequences and 111 ST8 sequences that were described previously.

All variant sites were extracted using snp-sites v2.3.3, and RaxML was used to estimate a maximum-likelihood phylogeny of all 292 acquired-region sequences, using a GTR model with gamma correction for among site rate variation and replicated for 100 bootstraps, after recombination was masked using Gubbins as previously described. This was outgroup rooted to the corresponding region of the ST8 reference sequence. A maximum-likelihood phylogeny was also estimated for the backbone region sequences, which was outgroup rooted to the corresponding region of the ST30 reference sequence.

The acquired region of the 96 ST239 sequences and the closest related non-ST239 clade, consisting of six ST30 isolates, were combined into a multiple sequence alignment. Variant sites were extracted using snp-sites v2.3.3. To calculate the time to the MRCA, Bayesian phylogenetic analysis was carried out using BEAST version 1.10.4, as previous (for the GTR nucleotide substitution model with all combinations of the strict and uncorrelated relaxed molecular clock models and constant- and exponential-growth models). This was repeated for the backbone region of the 96 ST239 sequences and the closest related non-ST239 clade, consisting of 18 ST8 isolates (one ST8 sequence was removed from the analysis, since it had no associated date of isolation). In both cases, the Bayes Factor showed no significant difference in the likelihood of the different models, and therefore the estimated time to the MRCA from the simplest model (strict molecular clock, constant population size) was recorded.

### Visualization of recombination in ST239.

ClonalFrameML v1.0-20 was used to estimate regions of recombination from the ST239 phylogeny, before recombination was masked. Ten isolates from the TW20-like clade were excluded from this analysis due to high sequence similarity to the ST239 reference sequence.

### Estimating evolutionary rates of the acquired and backbone regions.

The evolutionary rates of the acquired (minus the SCC*mec* element) and backbone regions of ST239 were estimated using BEAST analysis on 5,353 variant sites from the 96 ST239 backbone region sequences, and 1,449 variant sites from the ST239 acquired region sequences. The GTR nucleotide substitution model was used with all combinations of the strict and uncorrelated relaxed molecular clock models and constant and exponential growth models, as previously described.

### McDonald-Kreitman comparison of ST239, ST30, and ST8 core genes.

All 1,980 core genes from the 96 ST239 genomes, 111 ST8 genomes, and 57 ST30 genomes were converted into amino acid sequence alignments. Each of the ST239, ST30, and ST8 consensus core gene sequences that were generated previously using EMBOSS (84) was also converted into consensus amino acid sequences.

The number of fixed synonymous and fixed nonsynonymous SNPs was calculated for core genes within the whole ST239 genome, core genes within the backbone region, and core genes within the acquired region using snp-sites v2.3.3. This analysis was repeated for the 111 ST8 genomes collection and the 57 ST30 genomes collection. The McDonald-Kreitman neutrality index (N) was calculated as N = (P_n_/P_s_)/(D_n_/D_s_), where N is the net neutrality index, P_n_ is the number of nonsynonymous polymorphisms, P_s_ is the number of synonymous polymorphisms, D_n_ is the number of nonsynonymous substitutions, and D_s_ is the number of synonymous substitutions.

### Competition experiments.

Cryostocks of the 15 isolates were streaked on tryptic soy agar (TSA) and incubated at 37°C for 24 h. Single colonies were incubated for 24 h in 3 mL of TSB at 37°C with 225-rpm shaking. One ml of each culture was combined into a single mixture and mixed thoroughly. Genomic DNA was extracted and purified from 1 mL of the mixture, and sequencing was carried out, as previously described.

A portion (6 mL) of the mixture was pelleted, washed three times in phosphate-buffered saline (PBS), and separated into six 1-mL aliquots. These were then diluted 50× in either TSB, BHI, or PS. A total of 3 mL of each mixed culture was incubated at 37°C with 225-rpm shaking for 24 h. After 24 h, genomic DNA was extracted and purified, and sequencing was carried out, as previously described. All competition experiments were repeated in triplicate.

The DNA sequences from before and after each competition were mapped to the consensus sequence, as previously described. The number of reads supporting each unique variant site allele (for both the reference allele and the variant allele) was determined from the mapping of the raw sequence reads from each competition experiment. Any sites that were supported with a total of three reads or less, for both the variant allele and the consensus allele, were removed from the analysis to reduce the number of incorrect alleles due to sequencing error.

For each competition experiment, the number of supporting reads for each unique variant site was recorded. From this, the average coverage of all variants that were unique to each isolate was determined, before and after being exposed to the competition conditions for 24 h, to determine how the proportion of each isolate changed during each competition. The limit of detection for each isolate was also calculated, as *M*_Z_ = 4/[(*N*_Z_* + N*_Z_′)/*V*], where *M* is the minimum detection limit for isolate *Z*, *N*_Z_ is the total number of reads in support of isolate *Z* at sites unique to isolate *Z*, *N*_Z_′ is the total number of reads in support of non-*Z* isolates at sites unique to isolate *Z*, and *V* is the number of variant sites that are unique to isolate *Z*. Any isolate with an average coverage that was lower than the limit of detection was called at the limit of detection for that isolate. Raw competitive ability was calculated as the difference in log relative abundance of each isolate before and after competition in each replicate. Raw competition values were then rescaled, such that the mean competitive ability in each culture medium was equal to zero. This small correction factor was used to account for the fact that the true final density of some isolates was below the minimal detection threshold.

### Sequencing DNA from isolates for competition experiments.

Cryostocks of the 15 isolates were streaked on TSA, followed by incubation at 37°C for 24 h. Single colonies were incubated for 24 h in 3 ml of TSB at 37°C with 225-rpm shaking. Genomic DNA was extracted and purified using the Qiagen DNeasy blood and tissue kit and, following the protocol for purification of bacterial or yeast DNA with enzymatic lysis, using QIAcube. Sequencing was carried out using an Illumina HiSeq4000 system with 150-bp paired-end reads by the Oxford Genomic Centre, Wellcome Trust Centre for Human Genetics, University of Oxford, Oxford, UK.

The sequences were *de novo* assembled into contigs using SPAdes v3.13.0 (85) and ordered against the ST239, ST30, or ST8 reference sequences using abacas v1.3.1 (86). Sequences were annotated using PROKKA or UniProt, as previously described. Using Roary, we defined core genes as genes that were shared among all 15 isolates. Unique variant alleles were defined for each isolate, which were identified in only a single isolate, using snp-sites v2.3.3 (79).

To verify the validity of each unique variant site, each DNA sequence was remapped to the consensus sequence using bwa and SAMtools (71). The number of reads supporting each unique variant site was extracted, and only unique variant sites that could be used to accurately identify a single isolate and that were supported with four or more reads were included as true unique variant sites.

### AMR profiles.

Cryostocks of the 15 isolates were streaked on TSA, followed by incubation at 37°C for 24 h. Single colonies were incubated for 24 h in 3 ml of MH2 at 37°C with 225-rpm shaking. Cultures were diluted 100× in MH2 broth (to a density of ∼1 × 10^6^ CFU/ml) according to the MicroNaut evaluation protocol for MicroNaut-S MRSA/GP. Then, 100 μl was added to each well of a MicroNaut-S MRSA/GP plate, followed by incubation for 18 h at 37°C with 225-rpm shaking, after which the OD_595_ was measured. AMR breakpoints were assessed according to EUCAST standards (87). This was repeated in triplicate for each isolate. Tests for ampicillin and penicillin always gave the same results; hence, we considered the result from these two antibiotics as a single test score.

## References

[1] MacLean RC, San Millan A. 2019. The evolution of antibiotic resistance. Science 365:1082–1083. doi:10.1126/science.aax3879. 31515374

[2] Holden MTG, Hsu L-Y, Kurt K, Weinert LA, Mather AE, Harris SR, Strommenger B, Layer F, Witte W, de Lencastre H, Skov R, Westh H, Zemlicková H, Coombs G, Kearns AM, Hill RLR, Edgeworth J, Gould I, Gant V, Cooke J, Edwards GF, McAdam PR, Templeton KE, McCann A, Zhou Z, Castillo-Ramírez S, Feil EJ, Hudson LO, Enright MC, Balloux F, Aanensen DM, Spratt BG, Fitzgerald JR, Parkhill J, Achtman M, Bentley SD, Nübel U. 2013. A genomic portrait of the emergence, evolution, and global spread of a methicillin-resistant *Staphylococcus aureus* pandemic. Genome Res 23:653–664. doi:10.1101/gr.147710.112. 23299977 PMC3613582

[3] Nicolas-Chanoine M-H, Bertrand X, Madec J-Y. 2014. *Escherichia coli* ST131, an Intriguing Clonal Group. Clin Microbiol Rev 27:543–574. doi:10.1128/CMR.00125-13. 24982321 PMC4135899

[4] Wyres KL, Holt KE. 2016. *Klebsiella pneumoniae* population genomics and antimicrobial-resistant clones. Trends Microbiol 24:944–956. doi:10.1016/j.tim.2016.09.007. 27742466

[5] Wertheim HFL, Melles DC, Vos MC, van Leeuwen W, van Belkum A, Verbrugh HA, Nouwen JL. 2005. The role of nasal carriage in *Staphylococcus aureus* infections. Lancet Infect Dis 5:751–762. doi:10.1016/S1473-3099(05)70295-4. 16310147

[6] Pavillard R, Harvey K, Douglas D, Hewstone A, Andrew J, Collopy B, Asche V, Carson P, Davidson A, Gilbert G, Spicer J, Tosolini F. 1982. Epidemic of hospital‐acquired infection due to methicillin‐resistant *Staphylococcus aureus* in major Victorian hospitals. Med J Australia 1:451–454. doi:10.5694/j.1326-5377.1982.tb132413.x. 7099074

[7] Cookson BD, Phillips I. 1988. Epidemic methicillin-resistant *Staphylococcus aureus*. J Antimicrob Chemother 21:57–65. doi:10.1093/jac/21.suppl_c.57. 3384770

[8] Kerr S, Kerr GE, Mackintosh CA, Marples RR. 1990. A survey of methicillin-resistant *Staphylococcus aureus* affecting patients in England and Wales. J Hosp Infect 16:35–48. doi:10.1016/0195-6701(90)90047-R. 1974905

[9] Feil EJ, Nickerson EK, Chantratita N, Wuthiekanun V, Srisomang P, Cousins R, Pan W, Zhang G, Xu B, Day NPJ, Peacock SJ. 2008. Rapid detection of the pandemic methicillin-resistant *Staphylococcus aureus* clone ST 239, a dominant strain in Asian hospitals. J Clin Microbiol 46:1520–1522. doi:10.1128/JCM.02238-07. 18234867 PMC2292922

[10] Amorim ML, Faria NA, Oliveira DC, Vasconcelos C, Cabeda JC, Mendes AC, Calado E, Castro AP, Ramos MH, Amorim JM, de Lencastre H. 2007. Changes in the clonal nature and antibiotic resistance profiles of methicillin-resistant *Staphylococcus aureus* isolates associated with spread of the EMRSA-15 clone in a tertiary care Portuguese hospital. J Clin Microbiol 45:2881–2888. doi:10.1128/JCM.00603-07. 17626175 PMC2045296

[11] Conceição T, Aires-de-Sousa M, Füzi M, Tóth A, Pászti J, Ungvári E, van Leeuwen WB, van Belkum A, Grundmann H, de Lencastre H. 2007. Replacement of methicillin-resistant *Staphylococcus aureus* clones in Hungary over time: a 10-year surveillance study. Clin Microbiol Infect 13:971–979. doi:10.1111/j.1469-0691.2007.01794.x. 17697003

[12] Hu F, Zhu D, Wang F, Wang M. 2018. Current status and trends of antibacterial resistance in China. Clin Infect Dis 67:S128–S134. doi:10.1093/cid/ciy657. 30423045

[13] Robinson DA, Enright MC. 2004. Evolution of *Staphylococcus aureus* by large chromosomal replacements. J Bacteriol 186:1060–1064. doi:10.1128/JB.186.4.1060-1064.2004. 14762000 PMC344219

[14] Thomas JC, Godfrey PA, Feldgarden M, Robinson DA. 2012. Draft genome sequences of *Staphylococcus aureus* sequence type 34 (ST34) and ST42 hybrids. J Bacteriol 194:2740–2741. doi:10.1128/JB.00248-12. 22535928 PMC3347186

[15] Nimmo GR, Steen JA, Monecke S, Ehricht R, Slickers P, Thomas JC, Appleton S, Goering RV, Robinson DA, Coombs GW. 2015. ST2249-MRSA-III: a second major recombinant methicillin-resistant *Staphylococcus aureus* clone causing healthcare infection in the 1970s. Clin Microbiol Infect 21:444–450. doi:10.1016/j.cmi.2014.12.018. 25708549 PMC4564996

[16] Spoor LE, Richardson E, Richards AC, Wilson GJ, Mendonca C, RKr G, et al. 2015. Recombination-mediated remodelling of host-pathogen interactions during *Staphylococcus aureus* niche adaptation. Microb Genom 1. doi:10.1099/mgen.0.000036. PMC5320625 28348819

[17] Everitt RG, Didelot X, Batty EM, Miller RR, Knox K, Young BC, Bowden R, Auton A, Votintseva A, Larner-Svensson H, Charlesworth J, Golubchik T, Ip CLC, Godwin H, Fung R, Peto TEA, Walker AS, Crook DW, Wilson DJ. 2014. Mobile elements drive recombination hot spots in the core genome of *Staphylococcus aureus*. Nat Commun 5:3956. doi:10.1038/ncomms4956. 24853639 PMC4036114

[18] Chen L, Mathema B, Pitout JDD, DeLeo FR, Kreiswirth BN. 2014. Epidemic *Klebsiella pneumoniae* ST258 is a hybrid strain. mBio 5:e01355-14. doi:10.1128/mBio.01355-14. 24961694 PMC4073492

[19] Sheppard SK, Didelot X, Jolley KA, Darling AE, Pascoe B, Meric G, Kelly DJ, Cody A, Colles FM, Strachan NJC, Ogden ID, Forbes K, French NP, Carter P, Miller WG, McCarthy ND, Owen R, Litrup E, Egholm M, Affourtit JP, Bentley SD, Parkhill J, Maiden MCJ, Falush D. 2013. Progressive genome‐wide introgression in agricultural *Campylobacter coli*. Mol Ecol 22:1051–1064. doi:10.1111/mec.12162. 23279096 PMC3749442

[20] Brochet M, Rusniok C, Couvé E, Dramsi S, Poyart C, Trieu-Cuot P, Kunst F, Glaser P. 2008. Shaping a bacterial genome by large chromosomal replacements, the evolutionary history of *Streptococcus agalactiae*. Proc Natl Acad Sci USA 105:15961–15966. doi:10.1073/pnas.0803654105. 18832470 PMC2572952

[21] Monecke S, Slickers P, Gawlik D, Müller E, Reissig A, Ruppelt-Lorz A, et al. 2018. Molecular typing of ST239-MRSA-III from diverse geographic locations and the evolution of the SCCmec III element during its intercontinental spread. Front Microbiol 9:1436. doi:10.3389/fmicb.2018.01436. 30087657 PMC6066798

[22] Holden MTG, Lindsay JA, Corton C, Quail MA, Cockfield JD, Pathak S, Batra R, Parkhill J, Bentley SD, Edgeworth JD. 2010. Genome sequence of a recently emerged, highly transmissible, multi-antibiotic- and antiseptic-resistant variant of methicillin-resistant *Staphylococcus aureus*, sequence type 239 (TW). J Bacteriol 192:888–892. doi:10.1128/JB.01255-09. 19948800 PMC2812470

[23] Cong Y, Chan Y, Ragan MA. 2016. A novel alignment-free method for detection of lateral genetic transfer based on TF-IDF. Sci Rep 6:30308. doi:10.1038/srep30308. 27453035 PMC4958984

[24] Castillo-Ramírez S, Harris SR, Holden MTG, He M, Parkhill J, Bentley SD, Feil EJ. 2011. The impact of recombination on *dN/dS* within recently emerged bacterial clones. PLoS Pathog 7:e1002129. doi:10.1371/journal.ppat.1002129. 21779170 PMC3136474

[25] Botelho AMN, Cerqueira e Costa MO, Moustafa AM, Beltrame CO, Ferreira FA, Côrtes MF, Costa BSS, Silva DNS, Bandeira PT, Lima NCB, Souza RC, de Almeida LGP, Vasconcelos ATR, Narechania A, Ryan C, O’Brien K, Kolokotronis S-O, Planet PJ, Nicolás MF, Figueiredo AMS. 2019. Local diversification of methicillin-resistant *Staphylococcus aureus* ST239 in South America after its rapid worldwide dissemination. Front Microbiol 10:82. doi:10.3389/fmicb.2019.00082. 30873127 PMC6400870

[26] Petit RA, III, Read TD. 2018. *Staphylococcus aureus* viewed from the perspective of 40,000+ genomes. PeerJ 6:e5261. doi:10.7717/peerj.5261. 30013858 PMC6046195

[27] Harkins CP, Pichon B, Doumith M, Parkhill J, Westh H, Tomasz A, de Lencastre H, Bentley SD, Kearns AM, Holden MTG. 2017. Methicillin-resistant *Staphylococcus aureus* emerged long before the introduction of methicillin into clinical practice. Genome Biol 18:130. doi:10.1186/s13059-017-1252-9. 28724393 PMC5517843

[28] Baltrus DA. 2013. Exploring the costs of horizontal gene transfer. Trends Ecol Evol 28:489–495. doi:10.1016/j.tree.2013.04.002. 23706556

[29] San Millan A, MacLean RC. 2019. Fitness costs of plasmids: a limit to plasmid transmission, p 65–79. *In* Microbial transmission. American Society of Microbiology, Washington, DC. 10.1128/microbiolspec.mtbp-0016-2017 PMC11687550 28944751

[30] Brockhurst MA, Harrison E, Hall JPJ, Richards T, McNally A, MacLean C. 2019. The ecology and evolution of pangenomes. Curr Biol 29:R1094–R1103. doi:10.1016/j.cub.2019.08.012. 31639358

[31] Vogwill T, MacLean RC. 2015. The genetic basis of the fitness costs of antimicrobial resistance: a meta-analysis approach. Evol Appl 8:284–295. doi:10.1111/eva.12202. 25861386 PMC4380922

[32] Porse A, Schou TS, Munck C, Ellabaan MMH, Sommer MOA. 2018. Biochemical mechanisms determine the functional compatibility of heterologous genes. Nat Commun 9:522. doi:10.1038/s41467-018-02944-3. 29410400 PMC5802803

[33] Yang Q, Li M, Spiller OB, Andrey DO, Hinchliffe P, Li H, MacLean C, Niumsup P, Powell L, Pritchard M, Papkou A, Shen Y, Portal E, Sands K, Spencer J, Tansawai U, Thomas D, Wang S, Wang Y, Shen J, Walsh T. 2017. Balancing mcr-1 expression and bacterial survival is a delicate equilibrium between essential cellular defence mechanisms. Nat Commun 8. doi:10.1038/s41467-017-02149-0. PMC5727292 29233990

[34] Lee SM, Ender M, Adhikari R, Smith JMB, Berger-BäChi B, Cook GM. 2007. Fitness cost of staphylococcal cassette chromosome *mec* in methicillin-resistant *Staphylococcus aureus* by way of continuous culture. Antimicrob Agents Chemother 51:1497–1499. doi:10.1128/AAC.01239-06. 17283194 PMC1855501

[35] Knight GM, Budd EL, Whitney L, Thornley A, Al-Ghusein H, Planche T, Lindsay JA. 2012. Shift in dominant hospital-associated methicillin-resistant *Staphylococcus aureus* (HA-MRSA) clones over time. J Antimicrob Chemother 67:2514–2522. doi:10.1093/jac/dks245. 22761331

[36] Gray RR, Tatem AJ, Johnson JA, Alekseyenko AV, Pybus OG, Suchard MA, Salemi M. 2011. Testing spatiotemporal hypothesis of bacterial evolution using methicillin-resistant *Staphylococcus aureus* ST239 genome-wide data within a Bayesian framework. Mol Biol Evol 28:1593–1603. doi:10.1093/molbev/msq319. 21112962 PMC3115679

[37] Hsu L-Y, Harris SR, Chlebowicz MA, Lindsay JA, Koh T-H, Krishnan P, Tan T-Y, Hon P-Y, Grubb WB, Bentley SD, Parkhill J, Peacock SJ, Holden MT. 2015. Evolutionary dynamics of methicillin-resistant *Staphylococcus aureus* within a healthcare system. Genome Biol 16. doi:10.1186/s13059-015-0643-z. PMC4407387 25903077

[38] Harris SR, Feil EJ, Holden MTG, Quail MA, Nickerson EK, Chantratita N, Gardete S, Tavares A, Day N, Lindsay JA, Edgeworth JD, de Lencastre H, Parkhill J, Peacock SJ, Bentley SD. 2010. Evolution of MRSA during hospital transmission and intercontinental spread. Science 327:469–474. doi:10.1126/science.1182395. 20093474 PMC2821690

[39] Smyth DS, McDougal LK, Gran FW, Manoharan A, Enright MC, Song J-H, de Lencastre H, Robinson DA. 2010. Population structure of a hybrid clonal group of methicillin-resistant *Staphylococcus aureus*, ST239-MRSA-III. PLoS One 5:e8582. doi:10.1371/journal.pone.0008582. 20062529 PMC2797301

[40] Baines SL, Holt KE, Schultz MB, Seemann T, Howden BO, Jensen SO, van Hal SJ, Coombs GW, Firth N, Powell DR, Stinear TP, Howden BP. 2015. Convergent adaptation in the dominant global hospital clone ST239 of methicillin-resistant *Staphylococcus aureus*. mBio 6:e00080-15. doi:10.1128/mBio.00080-15. 25736880 PMC4358018

[41] Croucher NJ, Page AJ, Connor TR, Delaney AJ, Keane JA, Bentley SD, Parkhill J, Harris SR. 2015. Rapid phylogenetic analysis of large samples of recombinant bacterial whole-genome sequences using Gubbins. Nucleic Acids Res 43:e15. doi:10.1093/nar/gku1196. 25414349 PMC4330336

[42] Castillo-Ramírez S, Corander J, Marttinen P, Aldeljawi M, Hanage WP, Westh H, Boye K, Gulay Z, Bentley SD, Parkhill J, Holden MT, Feil EJ. 2012. Phylogeographic variation in recombination rates within a global clone of methicillin-resistant *Staphylococcus aureus*. Genome Biol 13:R126. doi:10.1186/gb-2012-13-12-r126. 23270620 PMC3803117

[43] Drummond AJ, Suchard MA, Xie D, Rambaut A. 2012. Bayesian phylogenetics with BEAUti and the BEAST 1.7. Mol Biol Evol 29:1969–1973. doi:10.1093/molbev/mss075. 22367748 PMC3408070

[44] Knox R. 1960. A new penicillin (BRL 1241) active against penicillin-resistant staphylococci. Br Med J 2:690–693. doi:10.1136/bmj.2.5200.690. 14410240 PMC2097972

[45] Bradley P, den Bakker HC, Rocha EPC, McVean G, Iqbal Z. 2019. Ultrafast search of all deposited bacterial and viral genomic data. Nat Biotechnol 37:152–159. doi:10.1038/s41587-018-0010-1. 30718882 PMC6420049

[46] van Tonder AJ, Mistry S, Bray JE, Hill DMC, Cody AJ, Farmer CL, Klugman KP, von Gottberg A, Bentley SD, Parkhill J, Jolley KA, Maiden MCJ, Brueggemann AB. 2014. Defining the estimated core genome of bacterial populations using a Bayesian decision model. PLoS Comput Biol 10:e1003788. doi:10.1371/journal.pcbi.1003788. 25144616 PMC4140633

[47] Robinson DA, Kearns AM, Holmes A, Morrison D, Grundmann H, Edwards G, O’Brien FG, Tenover FC, McDougal LK, Monk AB, Enright MC. 2005. Re-emergence of early pandemic *Staphylococcus aureus* as a community-acquired methicillin-resistant clone. Lancet 365:1256–1258. doi:10.1016/S0140-6736(05)74814-5. 15811459

[48] Didelot X, Wilson DJ. 2015. ClonalFrameML: efficient inference of recombination in whole bacterial genomes. PLoS Comput Biol 11:e1004041. doi:10.1371/journal.pcbi.1004041. 25675341 PMC4326465

[49] Nübel U, Dordel J, Kurt K, Strommenger B, Westh H, Shukla SK, Zemlicková H, Leblois R, Wirth T, Jombart T, Balloux F, Witte W. 2010. A timescale for evolution, population expansion, and spatial spread of an emerging clone of methicillin-resistant *Staphylococcus aureus*. PLoS Pathog 6:e1000855. doi:10.1371/journal.ppat.1000855. 20386717 PMC2851736

[50] Alam MT, Read TD, Petit RII, Boyle-Vavra S, Miller LG, Eells SJ, et al. 2015. Transmission and microevolution of USA300 MRSA in U.S. households: evidence from whole-genome sequencing. mBio 6:e00054-15. doi:10.1128/mBio.00054-15. 25759497 PMC4453535

[51] McAdam PR, Templeton KE, Edwards GF, Holden MTG, Feil EJ, Aanensen DM, Bargawi HJA, Spratt BG, Bentley SD, Parkhill J, Enright MC, Holmes A, Girvan EK, Godfrey PA, Feldgarden M, Kearns AM, Rambaut A, Robinson DA, Fitzgerald JR. 2012. Molecular tracing of the emergence, adaptation, and transmission of hospital-associated methicillin-resistant *Staphylococcus aureus*. Proc Natl Acad Sci USA 109:9107–9112. doi:10.1073/pnas.1202869109. 22586109 PMC3384211

[52] McDonald JH, Kreitman M. 1991. Adaptive protein evolution at the Adh locus in *Drosophila*. Nature 351:652–654. doi:10.1038/351652a0. 1904993

[53] Rand DM, Kann LM. 1996. Excess amino acid polymorphism in mitochondrial DNA: contrasts among genes from *Drosophila*, mice, and humans. Mol Biol Evol 13:735–748. doi:10.1093/oxfordjournals.molbev.a025634. 8754210

[54] Hershberg R, Petrov DA. 2010. Evidence that mutation is universally biased towards AT in bacteria. PLoS Genet 6:e1001115. doi:10.1371/journal.pgen.1001115. 20838599 PMC2936535

[55] Marvig RL, Sommer LM, Molin S, Johansen HK. 2015. Convergent evolution and adaptation of *Pseudomonas aeruginosa* within patients with cystic fibrosis. Nat Genet 47:57–64. doi:10.1038/ng.3148. 25401299

[56] Woods R, Schneider D, Winkworth CL, Riley MA, Lenski RE. 2006. Tests of parallel molecular evolution in a long-term experiment with *Escherichia coli*. Proc Natl Acad Sci USA 103:9107–9112. doi:10.1073/pnas.0602917103. 16751270 PMC1482574

[57] Gifford DR, Furió V, Papkou A, Vogwill T, Oliver A, MacLean RC. 2018. Identifying and exploiting genes that potentiate the evolution of antibiotic resistance. Nat Ecol Evol 2:1033–1039. doi:10.1038/s41559-018-0547-x. 29686236 PMC5985954

[58] Jevons MP, Parker MT. 1964. The evolution of new hospital strains of *Staphylococcus aureus*. J Clin Pathol 17:243–250. doi:10.1136/jcp.17.3.243. 14159451 PMC480732

[59] Ito T, Katayama Y, Asada K, Mori N, Tsutsumimoto K, Tiensasitorn C, Hiramatsu K. 2001. Structural comparison of three types of staphylococcal cassette chromosome *mec* integrated in the chromosome in methicillin-resistant *Staphylococcus aureus*. Antimicrob Agents Chemother 45:1323–1336. doi:10.1128/AAC.45.5.1323-1336.2001. 11302791 PMC90469

[60] Wales A, Davies R. 2015. Co-selection of resistance to antibiotics, biocides and heavy metals, and its relevance to foodborne pathogens. Antibiotics (Basel) 4:567–604. doi:10.3390/antibiotics4040567. 27025641 PMC4790313

[61] Andersson DI, Hughes D. 2010. Antibiotic resistance and its cost: is it possible to reverse resistance? Nat Rev Microbiol 8:260–271. doi:10.1038/nrmicro2319. 20208551

[62] Melnyk AH, Wong A, Kassen R. 2015. The fitness costs of antibiotic resistance mutations. Evol Appl 8:273–283. doi:10.1111/eva.12196. 25861385 PMC4380921

[63] Vogwill T, Kojadinovic M, MacLean RC. 2016. Epistasis between antibiotic resistance mutations and genetic background shape the fitness effect of resistance across species of *Pseudomonas*. Proc Biol Sci 283:20160151. doi:10.1098/rspb.2016.0151. 27170722 PMC4874708

[64] Hall AR, MacLean RC. 2011. Epistasis buffers the fitness effects of rifampicin-resistance mutations in *Pseudomonas aeruginosa*. Evolution 65:2370–2379. doi:10.1111/j.1558-5646.2011.01302.x. 21790582

[65] Scribner MR, Santos-Lopez A, Marshall CW, Deitrick C, Cooper VS. 2020. Parallel Evolution of tobramycin resistance across species and environments. mBio 11:e00932-20. doi:10.1128/mBio.00932-20. 32457248 PMC7251211

[66] Allen RC, Angst DC, Hall AR. 2018. Resistance gene carriage predicts growth of natural and clinical *Escherichia coli* isolates in the absence of antibiotics. Appl Environ Microbiol 85:e02111-18. doi:10.1128/AEM.02111-18. PMC6365833 30530714

[67] Nielsen KL, Pedersen TM, Udekwu KI, Petersen A, Skov RL, Hansen LH, Hughes D, Frimodt-Møller N. 2012. Fitness cost: a bacteriological explanation for the demise of the first international methicillin-resistant *Staphylococcus aureus* epidemic. J Antimicrob Chemother 67:1325–1332. doi:10.1093/jac/dks051. 22378682

[68] MacLean RC, Vogwill T. 2014. Limits to compensatory adaptation and the persistence of antibiotic resistance in pathogenic bacteria. Evol Med Public Health 2015:4–12. doi:10.1093/emph/eou032. 25535278 PMC4323496

[69] Planet PJ. 2017. Life after USA300: the rise and fall of a superbug. J Infect Dis 215:S71–S77. doi:10.1093/infdis/jiw444. 28375517 PMC5853207

[70] Song J-H, Hsueh P-R, Chung DR, Ko KS, Kang C-I, Peck KR, Yeom J-S, Kim S-W, Chang H-H, Kim Y-S, Jung S-I, Son JS, So T. M-k, Lalitha MK, Yang Y, Huang S-G, Wang H, Lu Q, Carlos CC, Perera JA, Chiu C-H, Liu J-W, Chongthaleong A, Thamlikitkul V, Van PH, ANSORP Study Group. 2011. Spread of methicillin-resistant *Staphylococcus aureus* between the community and the hospitals in Asian countries: an ANSORP study. J Antimicrob Chemother 66:1061–1069. doi:10.1093/jac/dkr024. 21393157

[71] Didelot X, Maiden MCJ. 2010. Impact of recombination on bacterial evolution. Trends Microbiol 18:315–322. doi:10.1016/j.tim.2010.04.002. 20452218 PMC3985120

[72] Morgulis A, Coulouris G, Raytselis Y, Madden TL, Agarwala R, Schäffer AA. 2008. Database indexing for production MegaBLAST searches. Bioinformatics 24:1757–1764. doi:10.1093/bioinformatics/btn322. 18567917 PMC2696921

[73] nh13/dwgsim. 2011. nh13/dwgsim. https://github.com/nh13/DWGSIM/wiki.

[74] Bolger AM, Lohse M, Usadel B. 2014. Trimmomatic: a flexible trimmer for Illumina sequence data. Bioinformatics 30:2114–2120. doi:10.1093/bioinformatics/btu170. 24695404 PMC4103590

[75] Li H, Durbin R. 2009. Fast and accurate short read alignment with Burrows-Wheeler transform. Bioinformatics 25:1754–1760. doi:10.1093/bioinformatics/btp324. 19451168 PMC2705234

[76] Kaya H, Hasman H, Larsen J, Stegger M, Johannesen TB, Allesøe RL, Lemvigh CK, Aarestrup FM, Lund O, Larsen AR. 2018. SCCmecFinder, a web-based tool for typing of staphylococcal cassette chromosome *mec* in *Staphylococcus aureus* using whole-genome sequence data. mSphere 3:e00612-17. doi:10.1128/mSphere.00612-17. 29468193 PMC5812897

[77] Jolley KA, Bray JE, Maiden MCJ. 2018. Open-access bacterial population genomics: BIGSdb software, the PubMLST.org website and their applications. Wellcome Open Res 3:124. doi:10.12688/wellcomeopenres.14826.1. 30345391 PMC6192448

[78] Hunt M, Mather AE, Sánchez-Busó L, Page AJ, Parkhill J, Keane JA, et al. 2017. ARIBA: rapid antimicrobial resistance genotyping directly from sequencing reads. Microbial Genomics 3. doi:10.1099/mgen.0.000131. PMC5695208 29177089

[79] Stamatakis A. 2014. RAxML version 8: a tool for phylogenetic analysis and post-analysis of large phylogenies. Bioinformatics 30:1312–1313. doi:10.1093/bioinformatics/btu033. 24451623 PMC3998144

[80] Seemann T. 2014. Prokka: rapid prokaryotic genome annotation. Bioinformatics 30:2068–2069. doi:10.1093/bioinformatics/btu153. 24642063

[81] Page AJ, Cummins CA, Hunt M, Wong VK, Reuter S, Holden MTG, Fookes M, Falush D, Keane JA, Parkhill J. 2015. Roary: rapid large-scale prokaryote pan genome analysis. Bioinformatics 31:3691–3693. doi:10.1093/bioinformatics/btv421. 26198102 PMC4817141

[82] Didelot X, Croucher NJ, Bentley SD, Harris SR, Wilson DJ. 2018. Bayesian inference of ancestral dates on bacterial phylogenetic trees. Nucleic Acids Res 46:e134. doi:10.1093/nar/gky783. 30184106 PMC6294524

[83] Page AJ, Taylor B, Delaney AJ, Soares J, Seemann T, Keane JA, et al. 2016. SNP-sites: rapid efficient extraction of SNPs from multi-FASTA alignments. Microb Genom 2:e000056. doi:10.1099/mgen.0.000056. 28348851 PMC5320690

[84] Rice P, Longden I, Bleasby A. 2000. EMBOSS: the European Molecular Biology Open Software Suite. Trends Genet 16:276–277. doi:10.1016/s0168-9525(00)02024-2. 10827456

[85] Bankevich A, Nurk S, Antipov D, Gurevich AA, Dvorkin M, Kulikov AS, Lesin VM, Nikolenko SI, Pham S, Prjibelski AD, Pyshkin AV, Sirotkin AV, Vyahhi N, Tesler G, Alekseyev MA, Pevzner PA. 2012. SPAdes: a new genome assembly algorithm and its applications to single-cell sequencing. J Comput Biol 19:455–477. doi:10.1089/cmb.2012.0021. 22506599 PMC3342519

[86] Assefa S, Keane TM, Otto TD, Newbold C, Berriman M. 2009. ABACAS: algorithm-based automatic contiguation of assembled sequences. Bioinformatics 25:1968–1969. doi:10.1093/bioinformatics/btp347. 19497936 PMC2712343

[87] European Committee on Antimicrobial Susceptibility Testing. 2014. Breakpoint tables for interpretation of MICs and zone diameters version 4.0. EUCAST, Växjö, Sweden. http://www.eucast.org.

[88] Sprouffske K, Wagner A. 2016. Growthcurver: an R package for obtaining interpretable metrics from microbial growth curves. BMC Bioinformatics 17. doi:10.1186/s12859-016-1016-7. PMC4837600 27094401

[89] Fuchs S, Mehlan H, Bernhardt J, Hennig A, Michalik S, Surmann K, Pané-Farré J, Giese A, Weiss S, Backert L, Herbig A, Nieselt K, Hecker M, Völker U, Mäder U. 2018. Aureo Wiki: the repository of the *Staphylococcus aureus* research and annotation community. Int J Med Microbiol 308:558–568. doi:10.1016/j.ijmm.2017.11.011. 29198880

