# Supplementary material for: Evolutionary Processes Driving the Rise and Fall of Staphylococcus aureus ST239, a Dominant Hybrid Pathogen
Source: mBio. 2021 Dec 14;12(6):e02168-21. doi: 10.1128/mBio.02168-21 (PMC8669471; doi:10.1128/mBio.02168-21)
Supplement: TABLE S1 [file mbio.02168-21-st001.pdf]

Supplementary Table 1A. Metadata of the ST239 sequences used in this study.

Sequence ID	GenBank Accession Number (Assembly) or Run Number (SRA)	Country of isolation	Continent of isolation	Date of isolation	Sequence data format	SNP divergence from ST239 reference	PubMed ID
CTXC01	GCA_001230325.1	Argentina	South America	1996	Assembly	5814	23270620
CTXW01	GCA_001229045.1	Argentina	South America	1996	Assembly	5418	23270620
CTXX01	GCA_001226505.1	Argentina	South America	1996	Assembly	4785	23270620
AGT120	ERR064919	Argentina	South America	1998	Short read	3760	NA
ERS640727	ERR732861	Australia	Oceania	1980	Short read	2710	25736880
ERS640783	ERR732875	Australia	Oceania	1982	Short read	2689	25736880
ERS640788	ERR732883	Australia	Oceania	1998	Short read	4834	25736880
ERS640754	ERR732893	Australia	Oceania	2002	Short read	4084	25736880
ERS640785	ERR732882	Australia	Oceania	2012	Short read	658	25736880
CP005288	GCA_000418345.1	Brazil	South America	1993	Assembly	6981	23873917
CP009681	GCA_000769575.1	Brazil	South America	1996	Assembly	6435	27152133
CP012011	GCA_001515745.1	Brazil	South America	2001	Assembly	6471	NA
LDPC01	GCA_001677355.1	Brazil	South America	2002	Assembly	7480	NA
CHL1	ERR006512	Chile	South America	1997	Short read	3766	20093474
CHL151	ERR006510	Chile	South America	1998	Short read	3873	20093474
LGWM01	GCA_002267325.1	Chile	South America	2012	Assembly	5172	28760895
CHI59	ERR064937	China	Asia	1998	Short read	3260	NA
CP007447	GCA_000709475.1	China	Asia	2004	Assembly	2138	239544
CP002643	GCA_000204665.1	China	Asia	2006	Assembly	4334	21551295
CP006838	GCA_000485885.1	China	Asia	2010	Assembly	4883	24309740
3HK	ERR006470	Czechia	Europe	2000	Short read	4097	20093474
2A8	ERR006540	Czechia	Europe	2001	Short read	3479	20093474
DEN907	ERR006514	Denmark	Europe	2001	Short read	665	20093474
CTWW01	GCA_001239465.1	Denmark	Europe	2006	Assembly	3530	23270620
CTXO01	GCA_001227085.1	Denmark	Europe	2009	Assembly	2966	NA
CTWS01	GCA_001229625.1	Egypt	Africa	2005	Assembly	5880	NA
ERS026791	ERR033471	France	Europe	2006	Short read	4009	NA
D8	ERR1213802	Gambia	Africa	2007	Short read	1071	27474712
GRE108	ERR064936	Greece	Europe	1998	Short read	60322	NA
GRE18	ERR064902	Greece	Europe	1998	Short read	4830	NA
GRE4	ERR064904	Greece	Europe	1998	Short read	5042	NA
GRE317	ERR064903	Greece	Europe	1999	Short read	4993	NA
HUSA304	ERR064928	Hungary	Europe	1993	Short read	3699	NA

HU106	ERR064927	Hungary	Europe	1996	Short read	3712	NA
HU109	ERR064909	Hungary	Europe	1996	Short read	5122	NA
HUR18	ERR064910	Hungary	Europe	1997	Short read	4666	NA
CTXS01	GCA_001224885.1	India	Asia	2006	Assembly	6834	NA
LWAK01	GCA_001652195.1	India	Asia	2014	Assembly	6279	NA
LWAM01	GCA_001652225.1	India	Asia	2014	Assembly	6648	NA
NESW01	GCA_002738565.1	India	Asia	2014	Assembly	11676	NA
MLQB01	GCA_001855085.1	India	Asia	2015	Assembly	6528	NA
CTXR01	GCA_001232225.1	Lithuania	Europe	1996	Assembly	5180	23270620
CTYA01	GCA_001239185.1	Lithuania	Europe	1996	Assembly	5444	23270620
CTYC01	GCA_001226905.1	Lithuania	Europe	1996	Assembly	5438	23270620
CTWZ01	GCA_001237525.1	Malaysia	Asia	1996	Assembly	1629	23270620
JQNU01	GCA_000787025.1	Malaysia	Asia	2003	Assembly	2310	NA
AMRE01	GCA_000313005.1	Malaysia	Asia	2009	Assembly	1899	23405328
ANPO01	GCA_000784385.1	Malaysia	Asia	2009	Assembly	2127	25197474
JKD6009	ERR732894	New Zealand	Oceania	2003	Short read	4714	25736880
CP002120	GCA_000145595.1	New Zealand	Oceania	2013	Assembly	5811	20802046
LGWY01	GCA_002267125.1	Peru	South America	2011	Assembly	6284	28760895
LGWZ01	GCA_002267145.1	Peru	South America	2011	Assembly	5747	28760895
MIHO01	GCA_002260265.1	Peru	South America	2013	Assembly	6203	28760895
CTXV01	GCA_001226265.1	Poland	Europe	1996	Assembly	7264	23270620
ERS026930	ERR038683	Poland	Europe	2006	Short read	4873	NA
ERS049893	ERR064932	Portugal	Europe	1990	Short read	3125	NA
ICP5014	ERR064934	Portugal	Europe	1993	Short read	3433	NA
HSJ216	ERR064920	Portugal	Europe	1998	Short read	3798	NA
HGSA142	ERR064922	Portugal	Europe	2003	Short read	4168	NA
CTXP01	GCA_001227985.1	Portugal	Europe	2005	Assembly	4737	23270620
CTXK01	GCA_001232765.1	Romania	Europe	2007	Assembly	6710	23270620
SRS1497005	SRR3658375	Russia	Europe	2011	Short read	4700	31329022
SRS1496939	SRR3658370	Russia	Europe	2013	Short read	4154	31329022
SRS2490451	SRR6003859	Russia	Europe	2016	Short read	4280	31329022
ERR053014	ERR053014	Singapore	Asia	1982	Short read	1252	25903077
ERR053016	ERR053016	Singapore	Asia	1985	Short read	310	25903077
ERR053031	ERR053031	Singapore	Asia	1997	Short read	370	25903077
ERR030243	ERR030243	Singapore	Asia	2002	Short read	499	25903077
ERR029473	ERR029473	Singapore	Asia	2009	Short read	334	25903077
LFED02	GCA_001184285.2	South Korea	Asia	2014	Assembly	994	27313284
CP013957	GCA_001641025.1	South Korea	Asia	2011	Assembly	1092	NA
CTXI01	GCA_001233925.1	Spain	Europe	1996	Assembly	6603	23270620
AHZL01	GCA_000401475.1	Switzerland	Europe	1990	Assembly	11802	NA
ERR064946	ERR064946	Thailand	Asia	2006	Short read	948	NA
CTWU01	GCA_001239585.1	Thailand	Asia	2007	Assembly	4380	23270620
ERR023864	ERR023864	Thailand	Asia	2008	Short read	1143	25491771

BPH0365	ERR732813	Turkey	Europe	1996	Short read	3329	25736880
CTWE01	GCA_001225725.1	Turkey	Europe	2006	Assembly	6223	23270620
CP012015	GCA_001515665.1	Turkey	Europe	2007	Assembly	6360	23270620
CTWD01	GCA_001226925.1	Turkey	Europe	2007	Assembly	6328	23270620
CTWI01	GCA_001236045.1	Turkey	Europe	2007	Assembly	6148	23270620
CTYZ01	GCA_001234465.1	Turkey	Europe	2008	Assembly	6286	23270620
CTWH01	GCA_001226645.1	Turkey	Europe	2009	Assembly	6457	23270620
FN433596	GCA_000027045.1	UK	Europe	2003	Assembly	0	19948800
ERR1212592	ERR1212592	UK	Europe	2009	Short read	2029	28348843
FFPZ01	GCA_900043625.1	UK	Europe	2010	Assembly	648	26672018
FGZX01	GCA_900058615.1	UK	Europe	2010	Assembly	1194	26672018
SRS1974492	SRR5251374	UK	Europe	2011	Short read	4477	29256859
URU34	ERR064925	Uruguay	South America	1997	Short read	3529	NA
URU110	ERR006556	Uruguay	South America	1998	Short read	3047	NA
JXZH02	GCA_001018635.2	USA	North America	1981	Assembly	4795	31346170
R35	ERR006550	USA	North America	1987	Short read	4102	NA
LHH1	ERR006548	USA	North America	1994	Short read	4436	NA
BK2421	ERR064899	USA	North America	1996	Short read	4027	NA
SR2852	SRR1177896	USA	North America	2005	Short read	754	24787619
CTXQ01	GCA_001234765.1	Vietnam	Asia	2004	Assembly	1949	23270620

Supplementary Table 1B. Metadata of the ST8 sequences used in this study.

Sequence ID	GenBank Accession Number (Assembly) or Run Number (SRA)	Country of isolation	Continent of isolation	Date of isolation	Sequence data format
ERR1535436	ERR1535436	Australia	Oceania	1989	Short read
ERR1535439	ERR1535439	Australia	Oceania	2003	Short read
ERR1535431	ERR1535431	Australia	Oceania	2008	Short read
ERR1535432	ERR1535432	Australia	Oceania	2010	Short read
ERR1535433	ERR1535433	Australia	Oceania	2011	Short read
CP007690	GCA_000695875.1	Belgium	Europe	1905	Assembly
ERR033306	ERR033306	Belgium	Europe	2006	Short read
ERR033347	ERR033347	Belgium	Europe	2006	Short read
CP019590	GCA_002088995.1	Canada	North America	2004	Assembly
ERR381019	ERR381019	Canada	North America	2008	Short read
ERR380998	ERR380998	Canada	North America	2009	Short read
ERR380994	ERR380994	Canada	North America	2010	Short read
ERR381026	ERR381026	Canada	North America	2011	Short read
CP007676	GCA_001045995.2	Colombia	South America	2006	Assembly
CP007672	GCA_001045795.2	Colombia	South America	2007	Assembly
CP007674	GCA_001021895.1	Colombia	South America	2007	Assembly
CP027101	GCA_001471715.2	Colombia	South America	2014	Assembly

ERR1588671	ERR1588671	Denmark	Europe	1957	Short read
ERR1588666	ERR1588666	Denmark	Europe	1968	Short read
ERR1712346	ERR1712346	Denmark	Europe	2004	Short read
ERR1712341	ERR1712341	Denmark	Europe	2007	Short read
SRR6169019	SRR6169019	Denmark	Europe	2016	Short read
SRR1955591	SRR1955591	France	Europe	1999	Short read
ERR033450	ERR033450	France	Europe	2006	Short read
ERR033462	ERR033462	France	Europe	2006	Short read
CP026961	GCA_001018725.2	French Guyana	South America	1996	Assembly
ERR1535412	ERR1535412	Gabon	Africa	2005	Short read
ERR1535387	ERR1535387	Gabon	Africa	2009	Short read
ERR1535352	ERR1535352	Gabon	Africa	2011	Short read
ERR1143478	ERR1143478	Gabon	Africa	2012	Short read
ERR1535423	ERR1535423	Gabon	Africa	2013	Short read
SRR4359408	SRR4359408	Germany	Europe	2001	Short read
CP003033	GCA_000245495.1	Germany	Europe	2002	Assembly
ERR1535365	ERR1535365	Germany	Europe	2006	Short read
ERR593213	ERR593213	Germany	Europe	2013	Short read
CP022290	GCA_002786465.1	Germany	Europe	2014	Assembly
ERR1535441	ERR1535441	Ghana	Africa	2005	Short read
ERR1535358	ERR1535358	Ghana	Africa	2011	Short read
ERR107788	ERR107788	Ireland	Europe	2001	Short read
ERR118522	ERR118522	Ireland	Europe	2001	Short read
ERR124443	ERR124443	Ireland	Europe	2004	Short read
ERR211899	ERR211899	Ireland	Europe	2004	Short read
ERR124475	ERR124475	Ireland	Europe	2010	Short read
ERR033701	ERR033701	Italy	Europe	2006	Short read
AP014921	GCA_002356675.1	Japan	Asia	2013	Assembly
SRR917579	SRR917579	Luxembourg	Europe	1997	Short read
SRR917586	SRR917586	Luxembourg	Europe	2005	Short read
ERR1535363	ERR1535363	Luxembourg	Europe	2006	Short read
SRR917585	SRR917585	Luxembourg	Europe	2007	Short read
SRR917652	SRR917652	Luxembourg	Europe	2010	Short read
ERR1143491	ERR1143491	Mozambique	Africa	2012	Short read
SRR3658373	SRR3658373	Russia	Europe	2013	Short read
SRR5100325	SRR5100325	Russia	Europe	2016	Short read
SRR5100330	SRR5100330	Russia	Europe	2016	Short read
ERR033296	ERR033296	Spain	Europe	2006	Short read
CP014362	GCA_003595365.1	Suriname	South America	2013	Assembly
CP014384	GCA_003595485.1	Suriname	South America	2013	Assembly
CP014444	GCA_002000865.1	Suriname	South America	2013	Assembly
ERR033573	ERR033573	Sweden	Europe	2006	Short read

ERR033574	ERR033574	Sweden	Europe	2006	Short read
SRR1786287	SRR1786287	Switzerland	Europe	2007	Short read
SRR1790318	SRR1790318	Switzerland	Europe	2007	Short read
ERR505339	ERR505339	Tanzania	Africa	2008	Short read
ERR505360	ERR505360	Tanzania	Africa	2008	Short read
ERR1143410	ERR1143410	Tanzania	Africa	2011	Short read
ERR505358	ERR505358	Tanzania	Africa	2013	Short read
ERR1213800	ERR1213800	The Gambia	Africa	2007	Short read
ERR1213806	ERR1213806	The Gambia	Africa	2008	Short read
ERR1712350	ERR1712350	The Netherlands	Europe	2005	Short read
ERR1712351	ERR1712351	The Netherlands	Europe	2006	Short read
ERR1712352	ERR1712352	The Netherlands	Europe	2007	Short read
SRR5029779	SRR5029779	UK	Europe	1935	Short read
ERR172082	ERR172082	UK	Europe	1998	Short read
ERR129281	ERR129281	UK	Europe	2003	Short read
ERR2072416	ERR2072416	UK	Europe	2012	Short read
SRR5651527	SRR5651527	UK	Europe	2015	Short read
SRR1103475	SRR1103475	USA, CA	North America	2011	Short read
KK095312	KK095312.1	USA, CA	North America	2012	Assembly
SRR1165956	SRR1165956	USA, CA	North America	2013	Short read
SRR3194930	SRR3194930	USA, CO	North America	2010	Short read
SRR3194933	SRR3194933	USA, CO	North America	2011	Short read
SRR4140011	SRR4140011	USA, FL	North America	2003	Short read
SRR4140097	SRR4140097	USA, FL	North America	2006	Short read
SRR4140090	SRR4140090	USA, FL	North America	2008	Short read
SRR4140057	SRR4140057	USA, FL	North America	2010	Short read
SRR4140079	SRR4140079	USA, FL	North America	2011	Short read
CP016855	GCA_001717645.3	USA, GA	North America	2010	Assembly
CP017094	CP017094.2	USA, GA	North America	2011	Assembly
CP025495	GCA_001735655.2	USA, GA	North America	2011	Assembly
SRR1786280	SRR1786280	USA, IL	North America	1965	Short read
SRR497495	SRR497495	USA, MA	North America	2003	Short read
KB820894	KB820894.1	USA, MA	North America	2007	Assembly
SRR1539657	SRR1539657	USA, MA	North America	2012	Short read
SRR5226531	SRR5226531	USA, MA	North America	2013	Short read
SRR3168638	SRR3168638	USA, MA	North America	2015	Short read
SRR3194883	SRR3194883	USA, MD	North America	2010	Short read
SRR3194942	SRR3194942	USA, MD	North America	2011	Short read
CP020619	GCA_002085525.1	USA, NE	North America	2013	Assembly
ERR134641	ERR134641	USA, NY	North America	2006	Short read
ERR302763	ERR302763	USA, NY	North America	2007	Short read
ERR338634	ERR338634	USA, NY	North America	2008	Short read

ERR134699	ERR134699	USA, NY	North America	2010	Short read
ERR134723	ERR134723	USA, NY	North America	2011	Short read
SRR3194889	SRR3194889	USA, OR	North America	2010	Short read
SRR3194948	SRR3194948	USA, OR	North America	2011	Short read
SRR3194899	SRR3194899	USA, PA	North America	2010	Short read
SRR3194910	SRR3194910	USA, TN	North America	2010	Short read
SRR3194949	SRR3194949	USA, TN	North America	2011	Short read
CP026076	GCA_001018685.2	USA, TX	North America	2002	Assembly
CP013231	GCA_001580515.1	USA, TX	North America	2013	Assembly
SRR4019282	SRR4019282	USA, TX	North America	2016	Short read

Supplementary Table 1C. Metadata of the ST30 sequences used in this study.

Sequence ID	GenBank Accession Number (Assembly) or Run Number (SRA)	Country of isolation	Continent of isolation	Date of isolation	Sequence data format
SRR1172654	SRR1172654	Argentina	South America	2013	Short read
LDIT01	GCA_001026765.1	Brazil	South America	2014	Assembly
ERR033374	ERR033374	Denmark	Europe	2006	Short read
ERR1753509	ERR1753509	Denmark	Europe	2013	Short read
ERR1753386	ERR1753386	Denmark	Europe	2015	Short read
ERR1592225	ERR1592225	DRC	Africa	2013	Short read
ERR1592254	ERR1592254	DRC	Africa	2013	Short read
ERR1592169	ERR1592169	DRC	Africa	2014	Short read
ERR1592321	ERR1592321	DRC	Africa	2015	Short read
ERR033635	ERR033635	Finland	Europe	2006	Short read
ERR033464	ERR033464	France	Europe	2006	Short read
ERR038658	ERR038658	France	Europe	2006	Short read
SRR4359400	SRR4359400	Germany	Europe	2002	Short read
ERR1143353	ERR1143353	Germany	Europe	2010	Short read
ERR1675778	ERR1675778	Germany	Europe	2014	Short read
ERR1195906	ERR1195906	Germany	Europe	2015	Short read
ERR033537	ERR033537	Italy	Europe	2006	Short read
ERR033542	ERR033542	Italy	Europe	2006	Short read
JHEC01	GCA_000708425.1	Jordan	Asia	2009	Assembly
LN626917	GCA_000953255.1	Kenya	Africa	2004	Assembly
JHEB01	GCA_000708405.1	Lebanon	Asia	2011	Assembly
ERR033698	ERR033698	Norway	Europe	2006	Short read
ERR038674	ERR038674	Norway	Europe	2006	Short read
ERR033550	ERR033550	Poland	Europe	2006	Short read
ERR038685	ERR038685	Poland	Europe	2006	Short read
ERR038688	ERR038688	Poland	Europe	2006	Short read
ERR033556	ERR033556	Portugal	Europe	2006	Short read

LWRA01	GCA_001639185.1	Russia	Europe	2013	Assembly
LWRC01	GCA_001639225.1	Russia	Europe	2014	Assembly
LWRD01	GCA_001639195.1	Russia	Europe	2014	Assembly
CP009554	GCA_000772025.1	South Korea	Asia	2014	Assembly
ERR033291	ERR033291	Spain	Europe	2006	Short read
ERR033578	ERR033578	Sweden	Europe	2006	Short read
ERR033589	ERR033589	Sweden	Europe	2006	Short read
ERR033590	ERR033590	Sweden	Europe	2006	Short read
ERR033593	ERR033593	Sweden	Europe	2006	Short read
AHZJ01	GCA_000401455.1	Switzerland	Europe	2005	Assembly
JXHZ01	GCA_000878025.1	Switzerland	Europe	2007	Assembly
SRR1786285	SRR1786285	Switzerland	Europe	2007	Short read
ERR1143398	ERR1143398	Tanzania	Africa	2011	Short read
ERR1143429	ERR1143429	Tanzania	Africa	2012	Short read
ERR505346	ERR505346	Tanzania	Africa	2012	Short read
ERR505389	ERR505389	Tanzania	Africa	2013	Short read
ERR829972	ERR829972	Tanzania	Africa	2014	Short read
ERR1213763	ERR1213763	The Gambia	Africa	2002	Short read
ERR1213777	ERR1213777	The Gambia	Africa	2004	Short read
ERR1213799	ERR1213799	The Gambia	Africa	2007	Short read
JYAJ02	GCA_001019455.2	UK	Europe	1935	Assembly
ERR109495	ERR109495	UK	Europe	1998	Short read
ERR129280	ERR129280	UK	Europe	2003	Short read
SRR5251295	SRR5251295	UK	Europe	2008	Short read
JPWO01	GCA_000739205.1	UK	Europe	2013	Assembly
ERR554777	ERR554777	USA	North America	2004	Short read
ERR554339	ERR554339	USA	North America	2009	Short read
CP017684	GCA_002633785.1	USA	North America	2011	Assembly
SRR1159198	SRR1159198	USA	North America	2012	Short read
SRR5822163	SRR5822163	USA	North America	2015	Short read

Supplementary Table 1D. Metadata of the experimental sequences used in this study. The reference genomes for the ST239, ST8 and ST30 isolates were NCBI accession numbers FN433596, CP007690 and NC002952, respectively.

Sequence ID	BEI Accession Number	Country of isolation	Continent of isolation	Date of isolation	SNP divergence from ST239, ST8 or ST30 reference
ST239A	NR-45889	Brazil	South America	1998	1295
ST239B	NR-45890	Brazil	South America	1999	1208
ST239C	TW20	UK	Europe	2003	0
ST239D	NR-50508	USA	North America	2011	250
ST8A	NCTC 8325	UK	Europe	1950	1525

ST8B	NR-45904	UK	Europe	1960	1436
ST8C	NR-45971	France	Europe	1997	1335
ST8D	NR-46214	USA	North America	2006	1533
ST8E	NR-49120	Brazil	South America	2012	1458
ST8F	JRH	UK	Europe	2016	N/A
ST30A	NR-46081	USA	North America	1996	2266
ST30B	NR-46024	France	Europe	2002	554
ST30C	NR-46067	UK	Europe	2002	135
ST30D	NR-46269	USA	North America	2005	275
ST30E	NR-46180	USA	North America	2006	325
