# Supplementary material for: Evolutionary Processes Driving the Rise and Fall of Staphylococcus aureus ST239, a Dominant Hybrid Pathogen
Source: mBio. 2021 Dec 14;12(6):e02168-21. doi: 10.1128/mBio.02168-21 (PMC8669471; doi:10.1128/mBio.02168-21)
Supplement: TABLE S2 [file mbio.02168-21-st002.pdf]

Supplementary Table 2A. BEAST model estimates of time to the MRCA of ST239.

**Substitution rate (95% HPD) (x10^{-6} SNPs/site/ year)**	**Region**	**BEAST evolutionary model (95% HPD intervals)**			
		**Strict clock Constant population size**	**Strict clock Exponential population size**	**Relaxed clock Constant population size**	**Relaxed clock Exponential population size**
	ST239 whole genome	**1.20** (1.13 - 1.28)	**1.20** (1.13 - 1.28)	**1.25** (0.99 - 1.53)	**1.29** (1.05 - 1.55)
	ST8-like region	**1.21** (1.13 - 1.29)	**1.21** (1.12 - 1.29)	**0.749** (0.494 - 1.08)	**1.25** (1.02 - 1.50)
	ST30-like region	**1.52** (1.32 - 1.71)	**1.48** (1.30 - 1.68)	**1.7** (1.31 -2.19)	**1.63** (1.27 -2.00)
**MRCA**	ST239 whole genome	**1940.1** (1934.7 - 1945.4)	**1940.1** (1934.6 - 1945.3)	**1929.1** (1899.3 - 1953.0)	**1946.5** (1931.5 - 1959.9)
	SCC*mec*-III	**1935.0** (1892.7 – 1967.6)	**1959.7** (1939.1 – 1976.4)	**1901.5** (1791.9 – 1968.3)	**1960.2** (1938.1 – 1977.4)

Supplementary Table 2B. BactDating model estimates of time to the MRCA of ST239.

	**BactDating evolutionary model (95% HPD)**		
**Estimated time to MRCA**	**Mixed gamma**	**Relaxed gamma**	**Strict gamma**
	1906.0 – 1949.8	1903.1 – 1947.3	1936.2 – 1943.0
