# Supplementary material for: Evolutionary Processes Driving the Rise and Fall of Staphylococcus aureus ST239, a Dominant Hybrid Pathogen
Source: mBio. 2021 Dec 14;12(6):e02168-21. doi: 10.1128/mBio.02168-21 (PMC8669471; doi:10.1128/mBio.02168-21)
Supplement: TABLE S3 [file mbio.02168-21-st003.pdf]

Supplementary Table 3. SCC*mec* prevalence in ST239 sequences in Staphopia. SCC*mec* elements with "some evidence" had some SCC*mec* gene sequences present (e.g., *ccrA* or *mecA*), but not enough to provide a definitive SCC*mec* type.

	**Number of ST239 sequences in Staphopia**	**Percentage of total ST239 sequences in Staphopia**
Evidence of SCC*mec*-III	2,979	93.7%
Some evidence of an SCC*mec* element	108	3.4%
No evidence of an SCC*mec* element	91	2.9%
Total	3,178	100%
