# Supplementary material for: Evolutionary Processes Driving the Rise and Fall of Staphylococcus aureus ST239, a Dominant Hybrid Pathogen
Source: mBio. 2021 Dec 14;12(6):e02168-21. doi: 10.1128/mBio.02168-21 (PMC8669471; doi:10.1128/mBio.02168-21)
Supplement: TABLE S4 [file mbio.02168-21-st004.pdf]

Supplementary Table 4A. Accession numbers of the ST30 genomes that share the closest common ancestor with the acquired region of the ST239 genome.

Accession Number	Date	Country	Source
SRR016399	1955	UK	Unknown
SRR016398	1955	UK	Clinical
SRR016780	1962	Australia	Unknown
SRR016388	1965	USA	Unknown
SRR016400	1968	USA	Blood
SRR2124638	Unknown	Unknown	Unknown

Supplementary Table 4B. Accession numbers of the ST8 genomes that share the closest common ancestor with the backbone region of the ST239 genome.

Accession Number	Date	Country	Source
ERR1588671	1957	Denmark	Bacteraemia
ERR1588666	1968	Denmark	Bacteraemia
ERR1712346	2004	Denmark	Human, clinical
ERR107788	2001	Ireland	Blood
ERR211899	2004	Ireland	Blood
ERR124443	2004	Ireland	Blood
ERR124475	2010	Ireland	Blood
ERR033574	2007	Sweden	Invasive human disease
SRR1786287	2007	Switzerland (Bern)	Food
ERR1535423	2017	Gabon	Unknown
ERR1213800	2007	Gambia	Skin and soft tissue disease
ERR1143491	2012	Mozambique	Human
ERR1535431	2008	Tanzania	Nasal carriage
ERR505339	2008	Tanzania	Nasal carriage
ERR505358	2013	Tanzania	Nasal carriage
SRR1786280	1965	USA (Chicago)	Human sample
KB820894	2007	USA (Boston)	Human sample
ERR338634	2008	USA (New York)	Soft tissue disease
ERR1007706	Unknown	Unknown	Unknown
