# Supplementary material for: Evolutionary Processes Driving the Rise and Fall of Staphylococcus aureus ST239, a Dominant Hybrid Pathogen
Source: mBio. 2021 Dec 14;12(6):e02168-21. doi: 10.1128/mBio.02168-21 (PMC8669471; doi:10.1128/mBio.02168-21)

Supplementary Figure 1. Midpoint rooted maximum-likelihood phylogeny of the SCC*mec*-III region of ST239. Units in total number of SNPs.

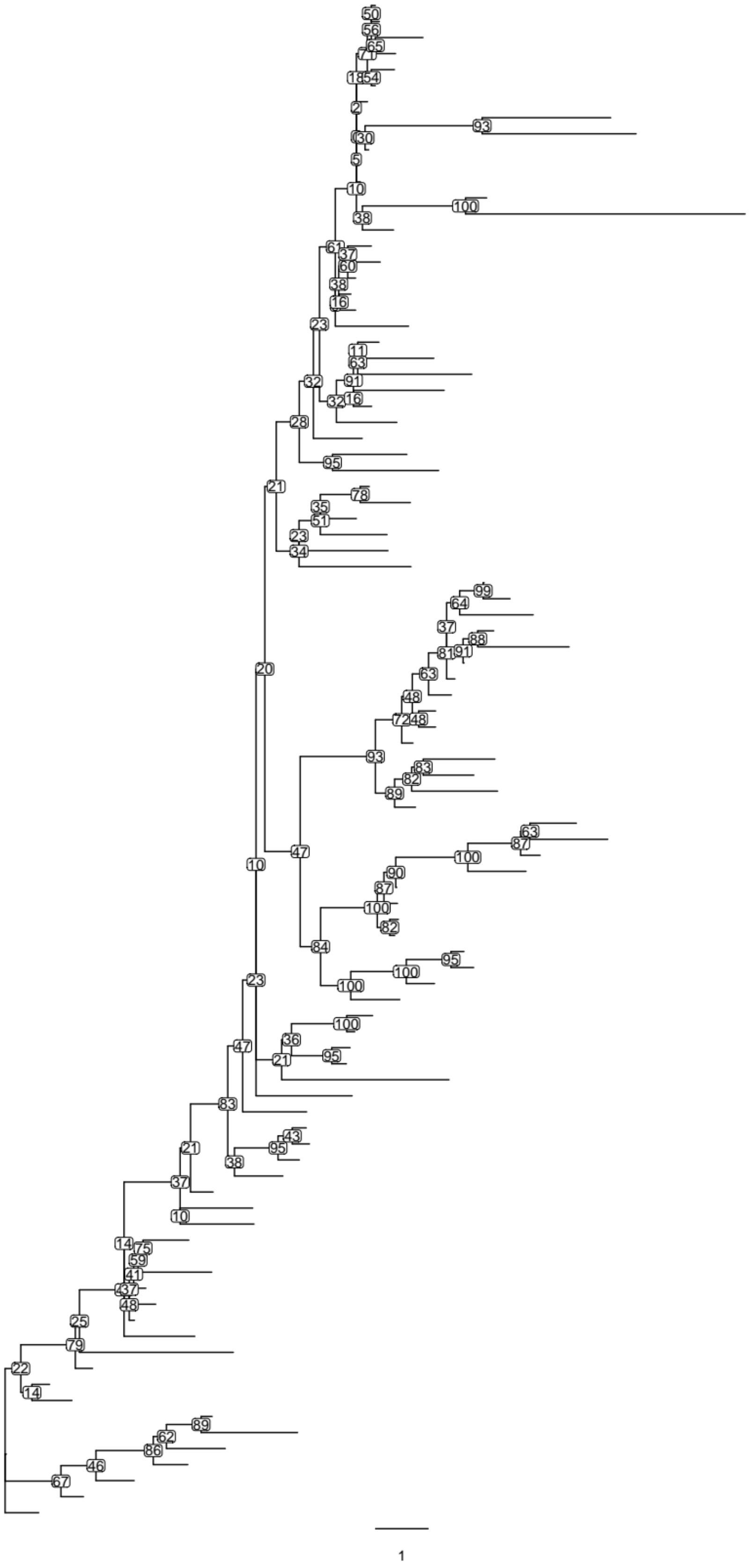

Supplement: FIG S1 [file mbio.02168-21-sf001.pdf]
