# Supplementary material for: Evolutionary Processes Driving the Rise and Fall of Staphylococcus aureus ST239, a Dominant Hybrid Pathogen
Source: mBio. 2021 Dec 14;12(6):e02168-21. doi: 10.1128/mBio.02168-21 (PMC8669471; doi:10.1128/mBio.02168-21)

Supplementary Figure 2. Number of nodes and tips affected by recombination, as estimated by ClonalFrameML at each position along the TW20 genome. Regions of high recombination are labelled 1, 2, 3 and 4.

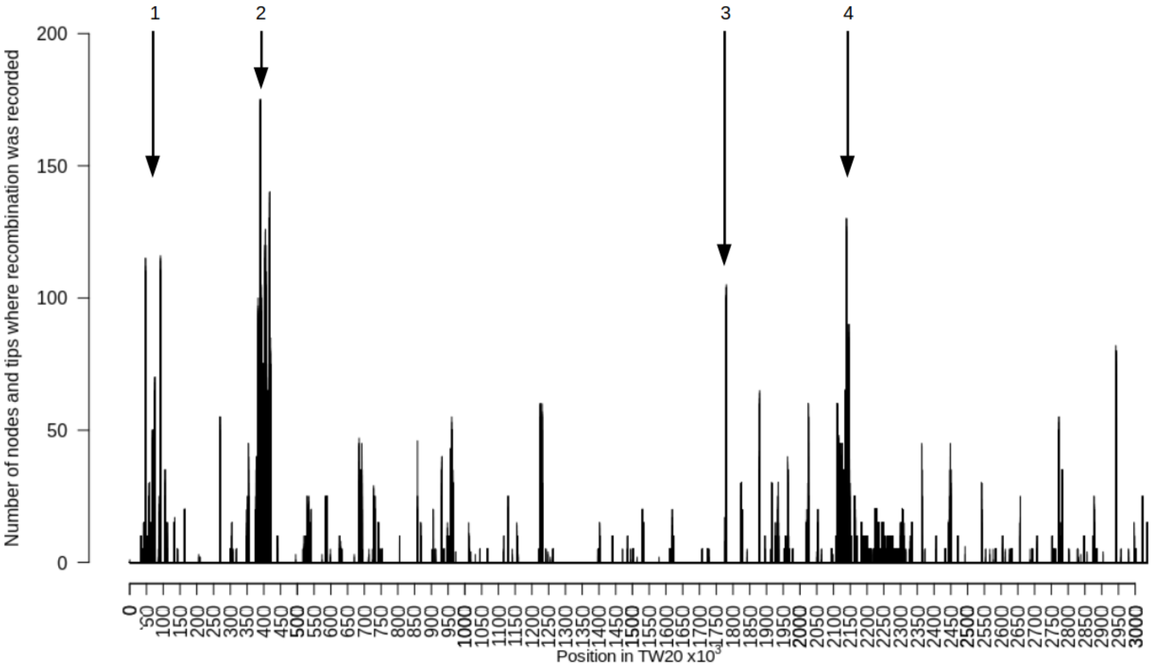

Supplement: FIG S2 [file mbio.02168-21-sf002.pdf]
