# Supplementary material for: Evolutionary Processes Driving the Rise and Fall of Staphylococcus aureus ST239, a Dominant Hybrid Pathogen
Source: mBio. 2021 Dec 14;12(6):e02168-21. doi: 10.1128/mBio.02168-21 (PMC8669471; doi:10.1128/mBio.02168-21)
Supplement: FIG S3 [file mbio.02168-21-sf003.pdf]

Supplementary Figure 3A. Schematic of unique variant site identification protocol. **3B.** Schematic of competition experimental protocol. **3C.** Schematic of protocol to estimate competitive ability.

A

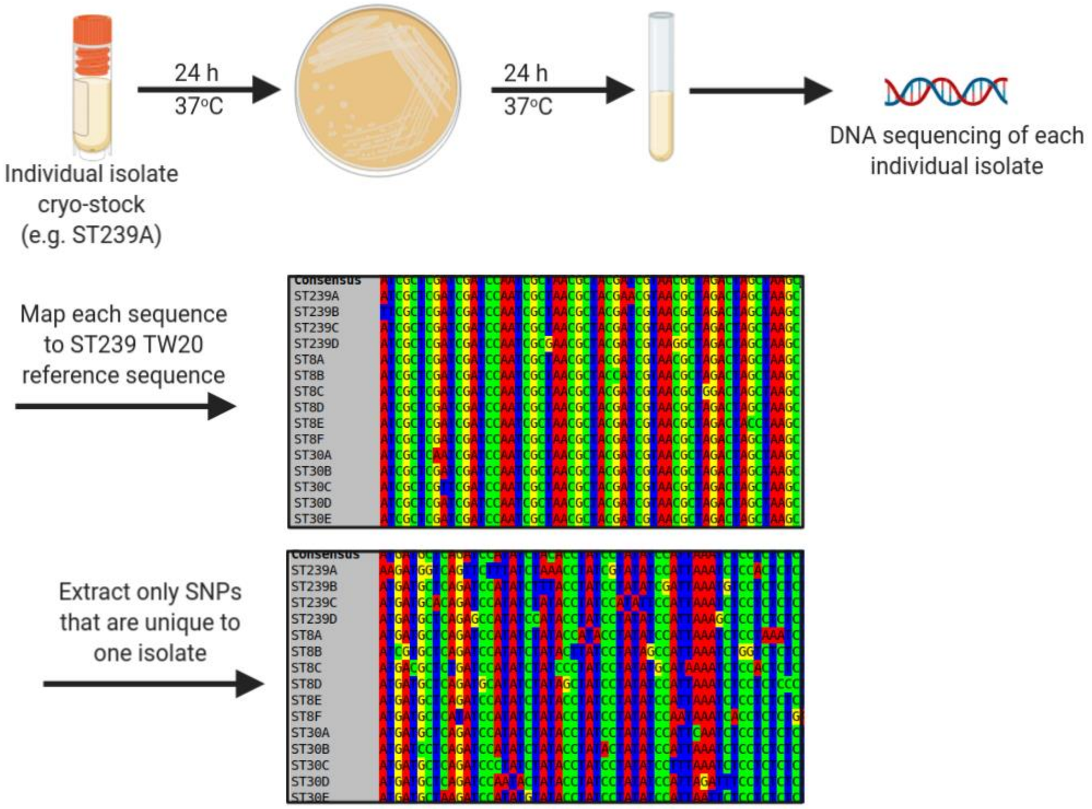

B

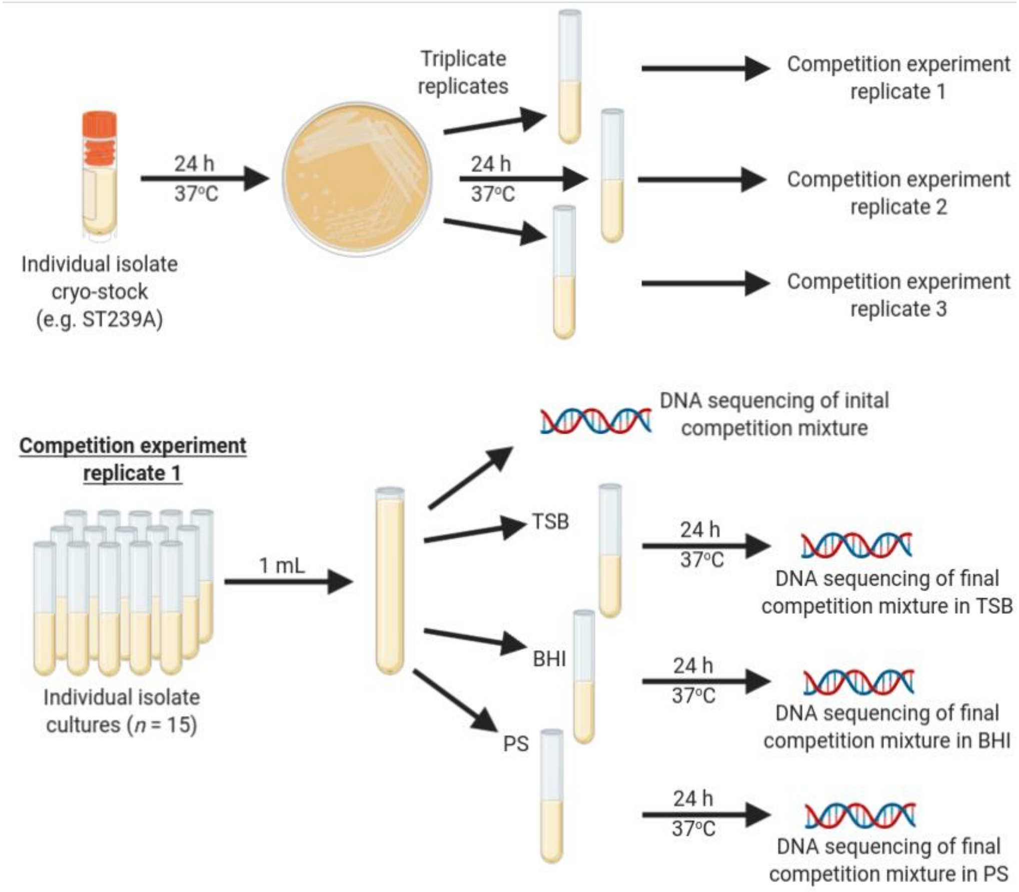

C

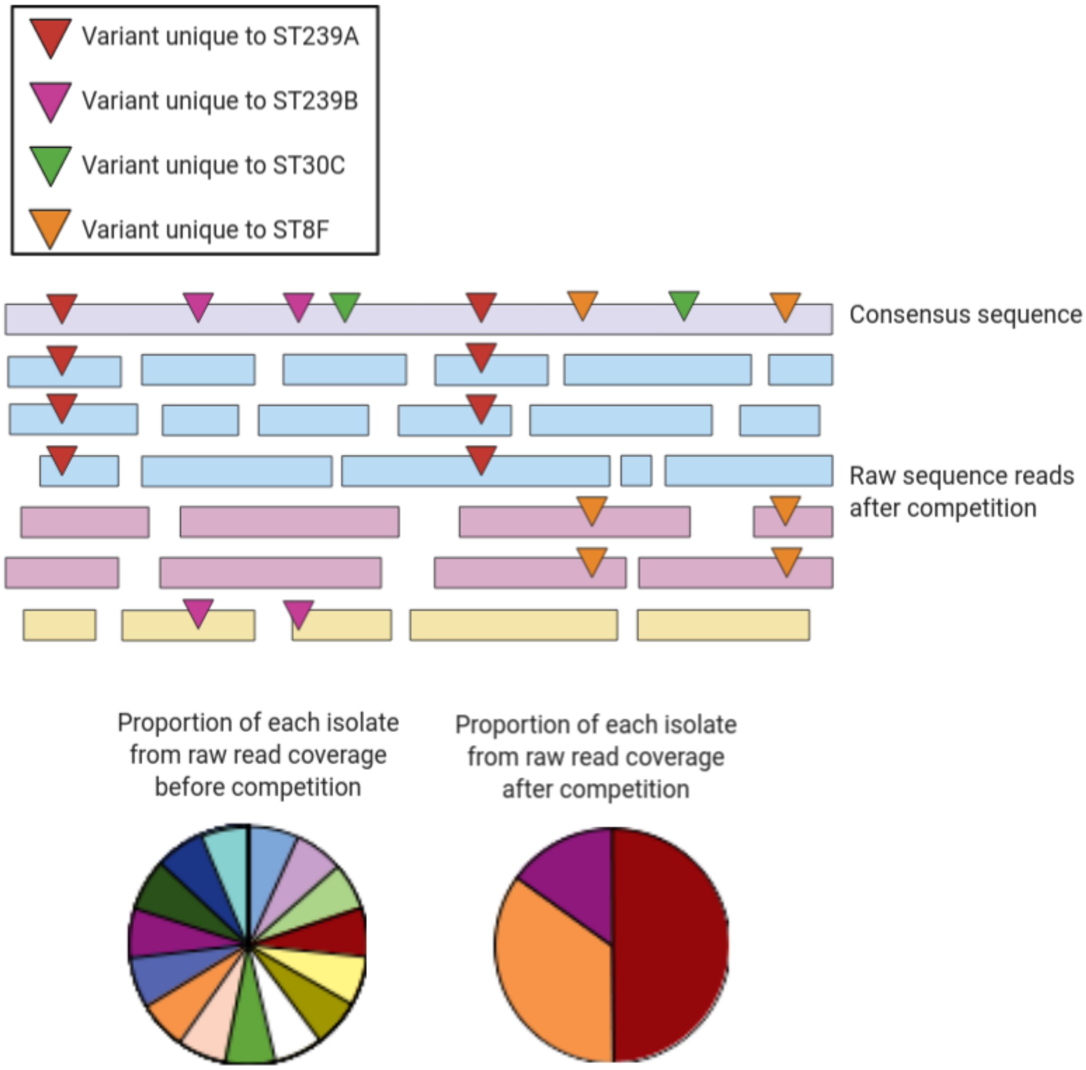
Variant unique to ST239A
Variant unique to ST239B
Variant unique to ST30C
Variant unique to ST8F
Consensus sequence
Raw sequence reads after competition
Proportion of each isolate from raw read coverage before competition
Proportion of each isolate from raw read coverage after competition
