# Supplementary material for: Evolutionary Processes Driving the Rise and Fall of Staphylococcus aureus ST239, a Dominant Hybrid Pathogen
Source: mBio. 2021 Dec 14;12(6):e02168-21. doi: 10.1128/mBio.02168-21 (PMC8669471; doi:10.1128/mBio.02168-21)
Supplement: TABLE S5 [file mbio.02168-21-st005.pdf]

Supplementary Table 5A. Reduced ANOVA table of significant competitive ability effects, with media effect removed.

Effect	df	Sum of squares	Mean squares	F-value	*p*-value
ST	2	0.2605196	0.1302598	7.692	0.00073
Media x ST	4	0.5271552	0.1317888	7.7823	0.00001
Isolate[ST]	12	4.2435429	0.353628575	20.88	$<1 \times 10^{-5}$
Error	134	6.9956092	0.0522060388	-	-

Supplementary Table 5B. Reduced ANOVA table of significant growth rate effects, with media effect removed.

Effect	df	Sum of squares	Mean squares	F-value	*p*-value
ST	2	1.1635513	0.58177565	4.9487	0.0080
Media	2	14.238509	7.119255	145.5380	$<1 \times 10^{-5}$
Media x ST	4	2.6565443	0.664136075	5.6493	0.0002
Isolate[ST]	12	3.1333904	0.261115867	2.2211	0.0120
Error	206(204)	24.217540	0.117560874	-	-
