# Supplementary material for: Evolutionary Processes Driving the Rise and Fall of Staphylococcus aureus ST239, a Dominant Hybrid Pathogen
Source: mBio. 2021 Dec 14;12(6):e02168-21. doi: 10.1128/mBio.02168-21 (PMC8669471; doi:10.1128/mBio.02168-21)
Supplement: FIG S4 [file mbio.02168-21-sf004.pdf]

**Supplementary Figure 4.** Growth rates (+/- s.e.; *N* = 9) of ST239, ST8 and ST30 isolates in TSB (A), BHI (B), PS (C). Panel D shows the mean (+/- s.e) growth rate of each ST across all three media. Both ST239 and ST30 have reduced growth rate relative to ST8 (ANOVA; $P < 0.05$). Cryostocks of the fifteen isolates were streaked on TSA, and incubated at 37$^{O}$C for 24 hours. Single colonies were incubated for 24 hours in 3 mL TSB at 37$^{O}$C with 225 RPM shaking. 1 mL of each culture was pelleted and washed three times in PBS, then diluted 50x in either TSB, BHI or PS. 100 µL of diluted culture was added to each well of a 96 well plate, in triplicate, and incubated at 37$^{O}$C with 225 RPM shaking. $OD_{595}$ was measured every 20 min over 24 hours, or until growth rate had reached plateau. This was repeated five times for each isolate. Growth curves were generated and analysed with Growthcurver[i] to calculate an exponential growth rate for each culture.

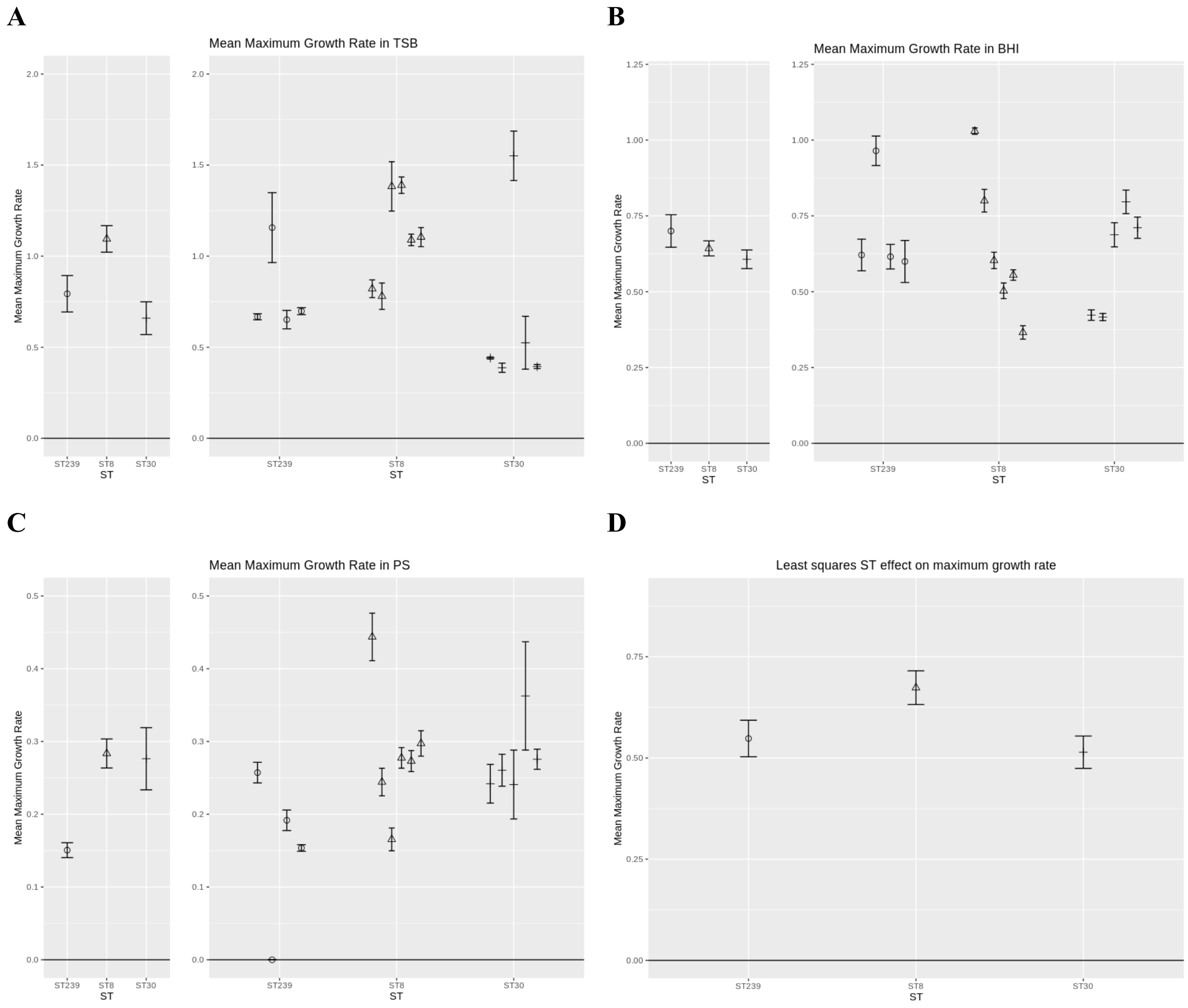


i Sprouffske K, Wagner A. Growthcurver: an R package for obtaining interpretable metrics from microbial growth curves. BMC Bioinformatics. 2016;17. doi:10.1186/s12859-016-1016-7
