# Supplementary material for: Evolutionary Processes Driving the Rise and Fall of Staphylococcus aureus ST239, a Dominant Hybrid Pathogen
Source: mBio. 2021 Dec 14;12(6):e02168-21. doi: 10.1128/mBio.02168-21 (PMC8669471; doi:10.1128/mBio.02168-21)
Supplement: TABLE S6 [file mbio.02168-21-st006.pdf]

Supplementary Table 6. Candidate genes in ST239 that show potential evidence of parallel evolution. Gene names and functions were identified in AureoWiki[ii].

Gene name	Genetic Region	No. SNPs	Function	General Function
walK	Acquired	13	Cell wall metabolism sensor histidine kinase WalK. Regulates genes involved in autolysis, biofilm formation and cell wall metabolism, and linked to vancomycin resistance.	AMR
spa	Acquired	62	Peptidoglycan-binding protein LysM. Virulence factor that enables host immune response evasion. Highly variable.	Virulence
gsiA	Acquired	13	Peptide ABC transporter ATP-binding protein. Down-regulation linked to antimicrobial peptide resistance.	AMR
ausA	Acquired	26	Hypothetical aureusimine synthesis protein – potential virulence factor.	Virulence
manP	Acquired	15	Phospho-transferase system mannose transporter subunit IIABC.	Metabolism
tarL	Acquired	25	Teichoic acid biosynthesis protein.	Metabolism
SAUPAN002689000	Backbone	11	Putative RNase adaptor protein RapZ.	Phage resistance
frp	Backbone	12	NAD(P)H-flavin oxidoreductase. Cell-wall protein linked to iron-restricted growth conditions.	Metabolism
grlA	Backbone	14	DNA topoisomerase IV subunit A. Fluoroquinolone resistance.	AMR
mprF	Backbone	10	Phosphatidylglycerol lysyltransferase, linked to methicillin resistance.	AMR
pknB	Backbone	10	Serine/threonine-protein kinase.	Metabolism
SAOUHSC_01130	Backbone	13	YfcC family arginine/ornithine APC transporter.	Metabolism
pycA	Backbone	11	Putative pyruvate carboxyl transferase.	Metabolism
glyA	Backbone	11	Serine hydroxymethyltransferase.	Metabolism
fdhA	Backbone	13	Formate dehydrogenase. Contains FeS cluster.	Metabolism
rpoB	Backbone	22	DNA-directed RNA polymerase subunit β. Rifampicin resistance.	AMR
ponA	Backbone	24	PBP2, cell wall biosynthesis. B-lactam-resistance.	AMR
sucA	Backbone	14	2-oxoglutarate dehydrogenase E1 component.	Metabolism
dinG	Backbone	14	ATP-dependent helicase.	Metabolism
dnaK	Backbone	14	Molecular chaperone.	Metabolism
pbpB	Backbone	15	Penicillin binding protein 2, related to β-lactam resistance.	AMR
atl	Backbone	17	Bifunctional autolysin.	Virulence
hsdS1	Backbone	14	Restriction endonuclease subunit S.	Metabolism
sdrH	Backbone	27	Hypothetical Serine-Aspartate Repeat protein. Highly variable.	Metabolism
lpdA	Backbone	9	Dihydrolipoamide dehydrogenase.	Metabolism
ybbH	Backbone	9	MurR/RpiR family transcriptional regulator.	Metabolism

ii Fuchs S, Mehlan H, Bernhardt J, Hennig A, Michalik S, Surmann K, et al. Aureo Wiki-The repository of the Staphylococcus aureus research and annotation community. International Journal of Medical Microbiology. 2018;308: 558–568. doi:10.1016/j.ijmm.2017.11.011
